# Stabilization of human interferon-α1 mRNA by its antisense RNA

**DOI:** 10.1007/s00018-012-1216-x

**Published:** 2012-12-08

**Authors:** Tominori Kimura, Shiwen Jiang, Mikio Nishizawa, Emi Yoshigai, Iwao Hashimoto, Masao Nishikawa, Tadayoshi Okumura, Hisao Yamada

**Affiliations:** 1 Laboratory of Microbiology and Cell Biology, Department of Pharmacy, College of Pharmaceutical Sciences, Ritsumeikan University, Kusatsu, Shiga 525-8577 Japan; 2 Laboratory of Medical Chemistry, Department of Biomedical Sciences, College of Life Sciences, Ritsumeikan University, Kusatsu, Shiga 525-8577 Japan; 3 Research Organisation of Science and Technology, Ritsumeikan University, Kusatsu, Shiga 525-8577 Japan; 4 Department of Surgery, Kansai Medical University, Moriguchi, Osaka 570-8506 Japan; 5 Department of Microbiology, Kansai Medical University, Moriguchi, Osaka 570-8506 Japan; 6 Department of Anatomy and Cell Science, Kansai Medical University, Moriguchi, Osaka 570-8506 Japan

**Keywords:** Interferon-α1 antisense RNA, mRNA stabilization, Interferon-α1 mRNA, microRNA, miR-1270, Regulatory RNA

## Abstract

Antisense transcription is a widespread phenomenon in the mammalian genome and is believed to play a role in regulating gene expression. However, the exact functional significance of antisense transcription is largely unknown. Here, we show that natural antisense (AS) RNA is an important modulator of interferon-α1 (IFN-α1) mRNA levels. A ~4-kb, spliced IFN-α1 AS RNA targets a single-stranded region within a conserved secondary structure element of the IFN-α1 mRNA, an element which was previously reported to function as the nuclear export element. Following infection of human Namalwa lymphocytes with* Sendai virus* or infection of guinea pig 104C1 fetal fibroblasts with *influenza virus* A/PR/8/34, expression of IFN-α1 AS RNA becomes elevated. This elevated expression results in increased IFN-α1 mRNA stability because of the cytoplasmic (but not nuclear) interaction of the AS RNA with the mRNA at the single-stranded region. This results in increased IFN-α protein production. The silencing of IFN-α1 AS RNA by sense oligonucleotides or over-expression of antisense oligoribonucleotides, which were both designed from the target region, confirmed the critical role of the AS RNA in the post-transcriptional regulation of IFN-α1 mRNA levels. This AS RNA stabilization effect is caused by the prevention of the microRNA (miRNA)-induced destabilization of IFN-α1 mRNA due to masking of the miR-1270 binding site. This discovery not only reveals a regulatory pathway for controlling *IFN*-*α1* gene expression during the host innate immune response against virus infection but also suggests a reason for the large number of overlapping complementary transcripts with previously unknown function.

**Electronic supplementary material:**

The online version of this article (doi:10.1007/s00018-012-1216-x) contains supplementary material, which is available to authorized users.

## Introduction

One of the greatest surprises of high-throughput transcriptome analysis has been the discovery that the mammalian genome is pervasively transcribed, producing many different and complex RNA families. In addition to a large number of alternative transcriptional start sites, termination and splicing patterns, a complex collection of new antisense, intronic, and intergenic transcripts has been found [1]. Moreover, almost half of the polyadenylated species are now known to be non-protein-coding RNAs. Although many studies have helped to unveil the function of many small non-coding RNAs, very little is known about the long non-coding (lncRNA) counterpart of the transcriptome [2].

Natural antisense transcripts (NATs) [3] are such lncRNA molecules, which are transcribed from the opposite strand of both protein-coding and non-coding genes. At least 1,000 pairs of sense-antisense transcripts are well conserved between mice and humans [4]. The diversity and extent of antisense transcription are changing the notion of NATs as transcriptional noise and are instead suggesting a pivotal role for antisense transcripts in eukaryotic gene expression (reviewed by [5, 6]). Thus, demonstrating a functional role for antisense transcripts in humans is intriguing.

Mammalian cells respond to virus infection by eliciting both innate and adaptive immune responses. One of the most effective innate antiviral responses is the production of type I interferons (IFNs) and the subsequent induction of IFN-stimulated genes. The pathways to and from type I IFNs, primarily IFN-α/β, are flexible. Families of sensors detect viral products and induce the expression of cytokines. One set of sensors is localized in the cytoplasm, while another set is localized in the plasma membrane and/or the membranes of organelles. Flexibility also exists in the signaling pathways used by IFN-α/β with the potential to induce the activation of multiple STAT (signal transducers and activators of transcription) molecules and their downstream targets (reviewed by [7]). While IFN-β is encoded by a single gene, IFN-α is encoded by a large family of structurally related genes localized in a cluster on human chromosome 9 [8]. Thirteen functional IFN-α genes (IFNAs) have been identified in humans and are reported to be induced in a coordinated fashion in virus-infected cells [9, 10].

Type I IFNs and their downstream products collectively limit viral replication and spread; therefore, IFN-α-based treatments are widely used for the treatment of chronic viral infections. Thus, a large number of studies of IFN regulation have focused on IFN-α protein expression and function, particularly transcriptional activation of type I IFN genes and the IFN-α signal transduction cascade. However, regulation at the RNA level has received less attention.

Here, we report that IFN-α1 antisense (AS) RNA, a NAT, is involved in determining post-transcriptional *IFNA1* expression. IFN-α1 AS RNA rapidly and reversibly upregulates IFN-α1 mRNA levels upon infection of human Namalwa B lymphocytes with* Sendai virus* (SeV; a paramyxovirus) and following infection of guinea pig (gp) 104C1 fetal fibroblasts with influenza virus A/PR/8/34 (PR8 virus). The data presented here show that the cytoplasmic sense–antisense interaction between the complementary transcripts results in stabilization of IFN-α1 mRNA. This effect was based on a novel regulatory role of IFN-α1 AS RNA, that of masking a miRNA-binding site in the mRNA. Therefore, IFN-α1 AS RNA is required to regulate IFN-α1 mRNA and, subsequently, IFN-α1 protein production. These data suggest that this previously unexamined AS RNA has a role in regulating *IFNA1* expression in a post-transcriptional manner and in driving host innate immunity against viral infection.

## Materials and methods

### Cell culture and virus propagation

Both human Namalwa B cells (B lymphocytes from Burkitt’s lymphoma; ATCC CRL-1432) and guinea pig 104C1 cells (fetal fibroblasts; ATCC CRL-1405) were maintained in RPMI-1640 medium supplemented with10 % heat-inactivated fetal calf serum (FCS) (R10). HeLa cells (ATCC CCL-2) were maintained in Dulbecco’s minimum essential medium containing 10 % FCS. MDCK (Madin-Darby canine kidney) cells (ATCC CCL-34) were maintained in Eagle’s minimum essential medium containing 10 % FCS. Both SeV and the mouse-adapted PR8 virus, influenza A/Puerto Rico/8/34 (A/PR/8/34, H1N1), were propagated in the allantoic cavity of 10-day-old embryonated hen eggs at 35 °C for 72 h (SeV) or at 35.5 °C for 48 h (PR8 virus). Allantoic fluid was pooled from multiple eggs, clarified by centrifugation, and frozen at −70 °C until use. The hemagglutination titer of each virus was determined by serial titration of virus stocks in eggs. The PR8 virus titer was measured with a plaque-forming assay using MDCK cells. IFN-α secreted into the culture supernatant of infected Namalwa cells was quantified using a human IFN-α ELISA kit according to the manufacturer’s instructions (Invitrogen, Carlsbad, CA, USA).

### Plasmids and recombinant lentivirus production

The human *IFNA1* AS nucleotide sequence, encoding a 1.1 kb exon that overlaps *IFNA1* and ends at nt 1 of the gene but in the opposite strand (see below), and the downstream fragment of the *IFNA1*-3′ untranslated region (UTR) (DS: nt 1–188 from the polyadenylation site of *IFNA1*) were amplified by the polymerase chain reaction (PCR; see below) using Namalwa cell genomic DNA as a template. ED7, CSS^+^ (conserved secondary structure), CSS and stem loop 2 (SL2) fragments [11], and 5′ UTR as well as 3′ UTR fragments of human *IFNA1* (DDBJ/EMBL/GenBank Accession Number: AB578886) were amplified by PCR using a full-length gene expression plasmid, phuIFN-α1, as a template [12]. The gene-specific primers used are listed in Supplementary Table 1. The amplicons and phuIFN-α1 plasmid were digested with *Hind*III/*Xba*I and cloned into the *Xba*I/*Hind*III sites of pCG-Bl [13] to generate phuIFN-α1 AS exon1.1, phuIFN-α1/mRNA revertant (mRNAR), /ED7R, /CSS^+^R, /CSSR, /SL2R, /5′UTRR, /3′UTRR, and /DSR expression plasmids, respectively. To construct the phuIFN-α1/bulged-stem loop (BSL) [11] revertant (BSLR) expression plasmid, 5′-phosphorylated primers, BSL/F and BSL/R, were annealed and cloned into the *Xba*I/*Hind*III sites of pCG-Bl (see Supplementary Table 1). Human chromosome 9 fragments containing *IFNA2*, *IFNA5*, and *IFNA17*, respectively, were amplified by PCR using gene-specific primer pairs (see Supplementary Table 1) and Namalwa cell genomic DNA as template. Each DNA fragment was then digested with *Hind*III/*Xba*I and cloned into the *Hind*III/*Xba*I sites of pSI [12].

The stem loop sequences of *Homo sapiens* micro-RNA (miR)-1270, -1287 and -483 (DDBJ/EMBL/GenBank Accession Numbers: MI0006407, MI0006349 and MI0002467, respectively) were retrieved from the miRBase database [14]. DNA fragments that contain 100 bases of both upstream and downstream native flank sequence of each miRNA stem loop were then amplified by PCR (see below) and cloned into the *Bam*HI and *Nhe*I sites of the pEGFP-miR expression vector (Cell Biolabs, San Diego, CA, USA) to create pEGFP-miR-1270, -miR-1287, and -miR-483. Details of the primer sequences and locations within the corresponding genes are shown in Supplementary Table 1. pLKO-miR-1270 lentiviral vector was then constructed by replacing the blunted *Xho*I and *Kpn*I fragment of shTORC2 (a kind gift from Fujisawa [15]) containing U6 promoter-shTORC2 complementary DNA (cDNA) with a blunted *Cla*I and *Kpn*I fragment of pEGFP-miR-1270 containing EF-1α promoter-miRNA-1270 or the same fragment from the pEGFP-miR-null control vector (Cell Biolabs). Recombinant lentiviruses for miR-1270 and miR-null were generated in HEK293T cells (ATCC CRL-1126) by the co-transfection of pLKO-miR-1270 or pLKO-null with the packaging construct pCMV∆R8.2 [16] and pVSV-G [17].

### Transfection, virus infection, and lentiviral transduction

Namalwa cells were subjected to magnet-assisted transfection, as described previously [18]. Briefly, non-adherent Namalwa cells were mixed with magnet-assisted transfection S (IBA, Goettingen, Germany) reagent and incubated on a magnetic plate for 15 min. To silence the expression of IFN-α1AS RNA, sense oligodeoxynucleotides (seODNs), which were phosphorothioate-modified (Operon Biotechnologies, Tokyo, Japan), were mixed with magnet-assisted transfection A reagent and added to immobilized cells for transfection using the manufacturer’s suggested protocol (IBA). After a 20-min incubation on the magnetic plate at room temperature, the cells were transferred to a CO_2_ incubator for a further 6 h incubation at 37 °C. Alternatively, to over-express IFN-α1 AS RNA and its truncated variants, the immobilized cells were transfected with 0.5 μg each of phuIFN-α1 ASexon1.1, phuIFN-α1/mRNAR, /ED7R, /CSS^+^R, /CSSR, /SL2R, /BSLR, /5′UTRR, /3′UTRR, /DSR or the parental pCG-Bl plasmid as described above, except for a 20-h incubation at 37 °C. Over-expression of miR-1270, -1287, or -483 was performed by transfection of the immobilized Namalwa cells with 2.9 μg each of pEGFP-miR-1270, -miR-1287, or -miR-483. 0.1 μg of pSV-β-galactosidase control vector (Promega, Madison, WI, USA) was included in each transfection mixture to normalize transfection efficiencies. The Beta-Glo assay system was employed to quantify β-galactosidase activity (Promega). For transduction with the lentiviral vectors, viral supernatants were collected 48 h after transfection of HEK293T cells with pLKO-miR-1270 or pLKO-null and Namalwa cells were transduced with the recombinant lentiviruses. The transduced cells were then selected with puromycin (2.5 μg/ml; Sigma-Aldrich, St. Louis, MO, USA), and mixtures of 1,000–2,000 clones of each virus were used for subsequent analysis.

To examine the effect(s) of AHCC (active hexose correlated compound; Amino Up Chemical, Sapporo, Japan), an extract derived from fungi of the *Basidiomycete* family [19], on IFN-α1 AS RNA expression levels, Namalwa cells were maintained in R10 medium supplemented with the compound at 0.5 mg/ml and sub-cultured three times. Transfected or AHCC-treated cells were then washed and infected with 50 hemagglutination units of SeV/10^6^ cells for 60 min. Cells were washed and incubated again at 37 °C for various time periods before total cellular RNA isolation. The seODNs were designed according to the IFN-α1 mRNA sequence and included loops that might cross-hybridize with the loops of the AS transcript, as previously described [18]. These sequences and names are listed in Supplementary Table 1. Antisense oligoribonucleotides (asORNs) (GeneDesign, Osaka, Japan) were also employed to introduce the BSLR fragment of IFN-α1 AS RNA into Namalwa cells. The asORNs were designed to avoid Toll-like receptor (TLR)7/8-mediated immune effects by excluding the GU-rich motifs [20] and contained 2′-*O*-methylation modifications at each nucleotide position [21]. Their sequences and names are as follows (an M indicates 2′-*O*-methylation and an asterisk indicates a phosphorothioate bond): asORN, 5′-U(M*)G(M*)U(M*)G(M*)G(M*)U(M*)A(M*)A(M*)A(M*)G(M*)A(M*)G(M*)G(M*)U(M*)U(M*)G(M*)A(M*)A(M*)G(M*)A(M*) U(M*)C(M*)U(M*)G(M*)C(M)-3′ and nc (negative control) ORN, 5′-G(M*)A(M*)C(M*)A(M*)G(M*)G(M*)A(M*)G(M*)G(M*)A(M*)A(M*)G(M*)G(M*)A(M*)G(M*)A(M*)G(M*)A(M*)U(M*)U(M)-3′ (*IFNA1* nt 346–322 and 224–205, respectively). 104C1 cells were infected with the PR8 virus at a multiplicity of infection of 25.5. After a further 12–48 h incubation at 37 °C, infected cells were harvested to examine gpIFN-α1 mRNA/AS as described below.

### Isolation of total cellular, nuclear, and cytoplasmic RNA

Total cellular RNA was isolated from Namalwa or 104C1 cells using TRIzol (Invitrogen). Alternatively, total nuclear and cytoplasmic RNAs were extracted from Namalwa cells using a modified NP40 method as described previously [12]. Each RNA sample was further treated with TRIzol LS (Invitrogen). RNA samples were then treated with a TURBO DNA-free DNase I kit (Applied Biosystems, Carlsbad, CA, USA) as described previously [13]. Poly(A)^+^ and poly(A)^−^ RNA were fractionated with a PolyATract mRNA isolation system (Promega) according to the manufacturer’s instructions.

### Northern-blot analysis

Northern blotting was employed to identify the expression of IFN-α1 AS RNA in the Namalwa cells that were infected with SeV for 24 h. The sense and antisense RNA probes were designed to minimize non-specific hybridization against mRNAs following homology searches using the Basic Local Alignment Tool (BLAST; http://blast.ncbi.nlm.nih.gov/). The selected probe sequence for the detection of IFN-α1 AS RNA was *IFNA1*-*AS* nt 554–355. A gene fragment containing this sequence, which was flanked with *Bam*HI and *Bgl*II sites, was amplified by PCR (see below). Four copies of the *IFNA1*-*AS* fragment were subcloned in tandem downstream of the pMNT/∆f1 vector T7 promoter [22] in both orientations. The sense and antisense probes were in vitro transcribed with T7 RNA polymerase (Takara Bio, Otsu, Japan) and [α-^32^P]CTP (10 μCi/μl, 3,000 Ci/mmol; PerkinElmer NEN, Boston, MA, USA). Briefly, linearized plasmid DNA (0.5 μg), 20 units of ribonuclease inhibitor (Takara Bio), 12 μM of CTP and 0.5 mM each of ATP, GTP and UTP, [α-^32^P]CTP and 40 units of T7 RNA polymerase were combined and incubated at 37 °C for 30 min. A half unit of RNase-free DNase I (Promega) was subsequently added and incubated at 37 °C for 15 min. The probes were phenol/chloroform-extracted and precipitated. Ten μg of total cellular RNA isolated from either SeV or mock infected Namalwa cells, 24 h after viral infection or poly(A)^+^ or poly(A)^−^ RNA fractionated from the total cellular RNA (see below) were separated and transferred to a nylon membrane (Whatman, GE Health Care, Tokyo, Japan) and UV-crosslinked. The membranes were pre-hybridized with alkaline-denatured salmon sperm DNA and then probed with α-^32^P-labeled sense or antisense probe at 60 °C for 17.5 h. The membranes were then washed twice in 2× standard saline phosphate EDTA (SSPE) and 0.1 % sodium dodecyl sulfate (SDS) at room temperature for 15 min and twice in 0.2× SSPE and 0.1 % SDS at 42 °C for 30 min. The film was autoradiographed at −30 °C for 22.5 h for IFN-α1 AS RNA and for 3 h for IFN-α1 mRNA. Details of the primer sequences and locations within *IFNA1*-*AS* are shown in Supplementary Table 1.

### Rapid amplification of 5′-cDNA ends (RACE) analysis

To determine the nucleotide sequences of 5′ exons of IFN-α1 AS RNA, poly(A)^+^ RNA from Namalwa cells infected with SeV for 24 h was reverse-transcribed with a biotinylated F1 primer (see Supplementary Table 1) (GeneDesign). A second-strand cDNA was then synthesized [23]. Resultant biotin-cDNA was trapped by streptavidin-Dynabeads (Life Technologies, Carlsbad, CA, USA) and subjected to 5′ RACE with a cDNA PCR Library Kit (Takara Bio) [18]. Details of the primer and adaptor sequences and their locations within *IFNA1* are shown in Supplementary Table 1.

### Strand-specific reverse-transcription quantitative polymerase chain reaction (RT-qPCR)

First-strand synthesis was performed essentially as described previously [12]. DNase I-treated total cellular RNA or nuclear and cytoplasmic RNA fractions were annealed with strand-specific primers. cDNA was synthesized in both the presence and absence of 100 U ReverTra Ace (TOYOBO, Osaka, Japan) at 50 °C for 30 min. The cDNAs were then amplified by PCR in a DNA Engine PTC-0200G (Bio-Rad, Hercules, CA, USA) using the following default thermocycler program: 1 min of pre-incubation at 95 °C followed by 22–38 cycles of 15 s at 95 °C, 1 min at 72 °C (reducing the annealing temperature 0.3 °C per cycle), and 30 s at 72 °C. To detect IFN-α1 mRNA, the R2 primer for RT and the F2B/R2 primer pair for PCR were used. To detect IFN-α1 AS RNA, the F1 primer for RT and the F1B/R1 primer pair for PCR were used. For quantitative comparisons between mRNA and AS RNA levels, the above cDNAs were PCR-amplified with the F2B/R2 or F1B/R1 primer pairs. For the 18S rRNA internal control, a nona-deoxyribonucleotide random primer mixture (Takara Bio) for RT and the 18S F/R primer pair for PCR were used. For detection of gpIFN-α1 mRNA and AS, the gpR1 and gpF1 primers, respectively, for RT and the gpF1B/gpR1 primer pair for PCR were used. For gpβ-actin mRNA (an internal control), the nona-deoxyribonucleotide random primer mixture for RT and the gpβ-actin F/R primer pair for PCR were used. Details of the primer sequences and locations within the corresponding genes are shown in Supplementary Table 1. The primer pairs employed did not generate PCR products in the absence of RT. The RNA levels were measured in at least three independent experiments with triplicate samples by real-time PCR using SYBR Green I (Roche Diagnostics, Basel, Switzerland) in a Chromo 4 real-time PCR analysis system apparatus (Bio-Rad). At the end of each real-time PCR reaction, the cycle threshold was determined at the level that reflected the best kinetic PCR parameters [24], and melting curves were acquired and analyzed to confirm that all primer pairs generated single products. The copy numbers of IFN-α1 mRNA/AS RNA and gpIFN-α1 mRNA/AS RNA were normalized to 18S rRNA and gpβ-actin mRNA, respectively. They are presented as “relative RNA expression”, which indicates the fold change of each RNA copy relative to that at 0 h after SeV or PR8 virus infection of control cells. Alternatively, ten-fold dilutions of a known concentration of phuIFN-α1 (30–3 × 10^5^ copies) or phuIFN-α1 AS (10–10^5^ copies) were assayed in the same run using the strand-specific qPCR described above for the samples shown in Fig. 3. The regression lines from each dilution curve were used to determine the copy numbers of IFN-α1 AS RNA or mRNA in each sample [25].

### Assessment of IFN-α1 mRNA/AS RNA PCR primer cross-reactivity among the IFN-α superfamily members

Alignment of IFN-α1 mRNA/AS RNA RT-PCR primer sequences was performed for *IFNA* gene family members using a function of GENETYX-MAC software (Ver. 15.0.0) (Genetyx Corporation, Tokyo, Japan).

### PCR mapping for characterization of IFN-α1 AS RNA

IFN-α1 AS RNA, extracted from SeV-infected Namalwa cells at 24 h post-infection, was reverse-transcribed as described above with either the RT2 or the F1 primer. The synthesized cDNAs were then PCR-amplified with the following primer pairs to localize the 5′ end of the IFN-α1 AS RNA: PCR1F and PCR1R, F1B and R1, PCR3F and PCR3R, PCR4F and PCR4R, or PCR5F and PCR5R. To further localize the 5′ end, IFN-α1 AS RNA at 24 h post-infection was reverse-transcribed with the RT2 primer and the cDNA was PCR-amplified with the following primer pairs: PCR4F and PCR9R, PCR10R, PCR11R, PCR12R, or PCR13R. To localize the 3′ end of IFN-α1 AS RNA, the following combinations of RT primer and PCR primer pair were used: RT1 for RT and RT1/PCR1R for PCR; RT-76 for RT and PCR7F/PCR7R for PCR; RT-199 or RT-498 for RT and PCR6F/PCR6R for PCR. Details of the primer sequences and locations within *IFNA1* and its flanking sequences are shown in Supplementary Table 1.

### Quantification of IFN-α1 mRNA half-lives

Actinomycin D (ActD; 0.75 or 2 μg/ml; Sigma-Aldrich) was added to phuIFN-α1*/*mRNAR- or S1-transfected Namalwa cells 6 or 12 h after SeV infection to block transcription. Cells were then harvested for total cellular RNA isolation at 0, 0.5, 1, 2, 3, and 4 h after the addition of ActD and analyzed to measure the half-lives of IFN-α1 mRNA by strand-specific RT-qPCR, as described above.

### Microinjections and RNA-fluorescence in situ hybridization (FISH)

Nuclear microinjections of HeLa cells and RNA-FISH were performed as previously described [12] using the phuIFN-α1/ED7 expression plasmid, a digoxigenin-labeled oligonucleotide probe and fluorescein isothiocyanate-conjugated antibodies. Visualization was performed with an Olympus Fluoview FV300 confocal laser scanning microscope.

### Statistical analysis and informatics

Results in the figures are a representative of at least three independent experiments with triplicate samples generating similar findings. Differences in Fig. 7 were analyzed using Student’s *t* test.

### Accession numbers

Gene accession numbers were deposited in DDBJ/EMBL/GenBank as follows: AB578886 (human *IFNA1*), AB578885 (human *IFNA1* AS), AB671739 (gp*IFNA1*) and JQ407016 (gp*IFNA10*). The sequence numbering presented in this report is based on the following DDBJ/EMBL/GenBank sequences: NM_000605, NM_021068, NM_002169, NM_021002, NM_021057, NM_002170, NM_002171, NM_006900, NM_002172, NM_002173, NM_021268 and NM_002175 for human *IFNA2*, *IFNA4*, *IFNA5*, *IFNA6*, *IFNA7*, *IFNA8*, *IFNA10*, *IFNA13*, *IFNA14*, *IFNA16*, *IFNA17*, and *IFNA21*, respectively. Gpβ-actin mRNA, complete cds is based on AF508792. The miRNA precursor genes employed in this study were hsa-miR-1270, -miR-1287, and -miR-483 (DDBJ/EMBL/GenBank sequences: MI0006407, MI0006349 and MI0002467, respectively).

## Results

### Identification of a naturally occurring antisense transcript overlapping human *IFNA1*

**Fig. 1:**
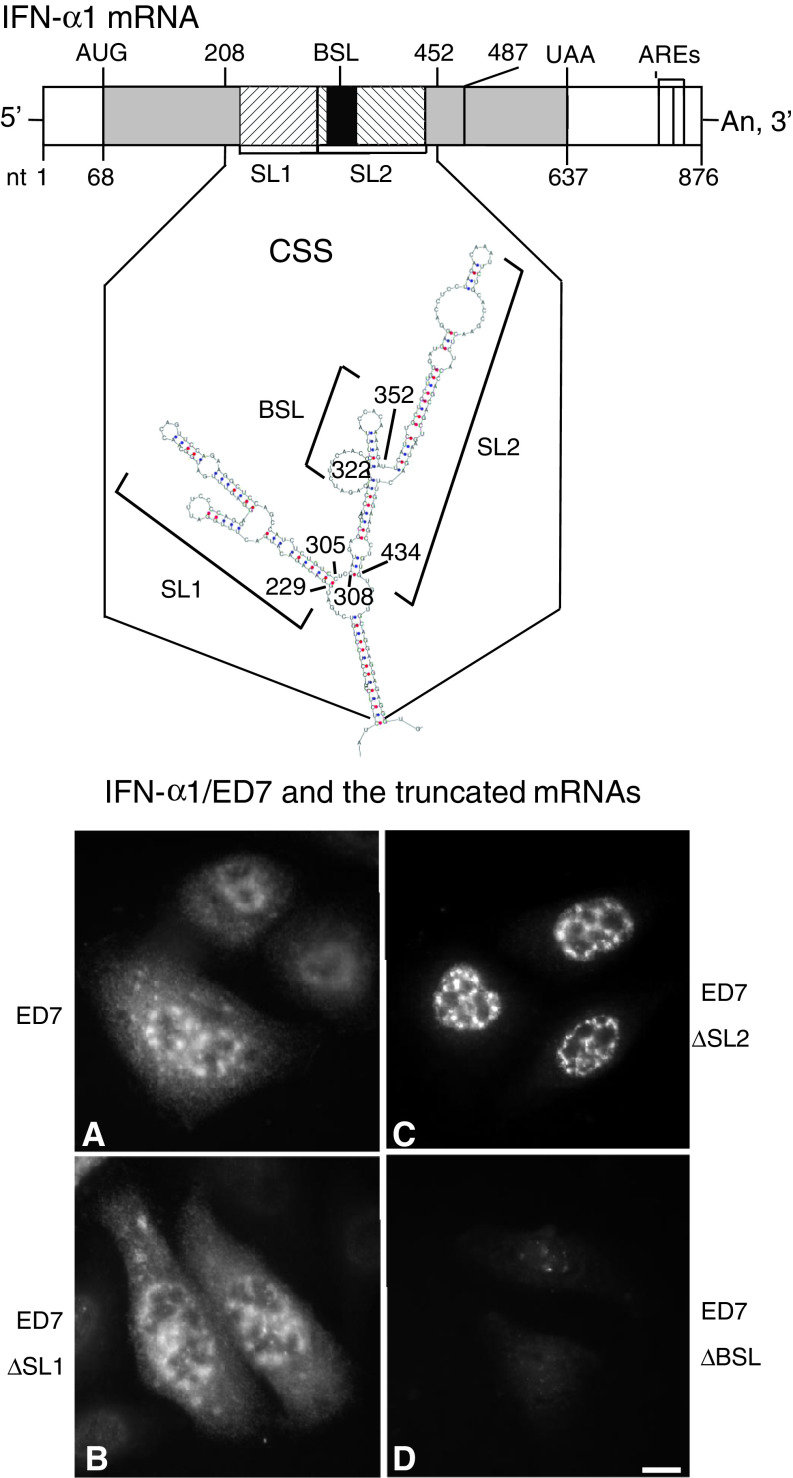
Truncation of the BSL from the CSS results in failure to detect IFN-α1 mRNA by fluorescence in situ hybridization. Results from a previous computational analysis of the folding potentials of the IFN-α1 mRNA export element, as well as internal deletion-mutagenesis and constitutive export assays, are shown. **a** IFN-α1/ED7 mRNA; **b** SL1 deletion mutant (ED7 ∆SL1); **c** SL2 deletion mutant (ED7 ∆SL2); **d** BSL deletion mutant (ED7 ∆BSL). CSS: nt 208–452, SL1: nt 229–305, SL2: nt 308–434, BSL: nt 322–352. The linear diagram shows IFN-α1 mRNA, which consists of the 5′ UTR (nt 1–67), CDS (protein-coding sequences; nt 68–637) and the 3′ UTR (nt 638–876). AREs indicate the adenylate uridylate-rich element motifs present in the 3′ UTR. *Bar* 10 μm. Figure reproduced with modifications from Kimura et al. [11] with permission

We recently reported that the coding region of human IFN-α1 mRNA contains a novel *cis*-active element that is responsible for the CRM1-dependent nuclear export of this specific message [11]. This element forms novel secondary structures, termed the conserved secondary structure (CSS), comprising two adjacent putative stable stem loop (SL) structures (Fig. 1; IFN-α1 mRNA nt 208–452). Internal deletion-mutagenesis and constitutive export assays of each SL structure, using IFN-α1/ED7 mRNA (nt 68–487; the shortest functional mRNA mutant), demonstrated that SL2 acts as a core element by conferring the export function on the CSS [11] (see also Fig. 1a–c). We also found that deletion of BSL structure in SL2 appeared to selectively impair the stability of IFN-α1/ED7 mRNA [11] (see also Fig. 1d).

**Fig. 2:**
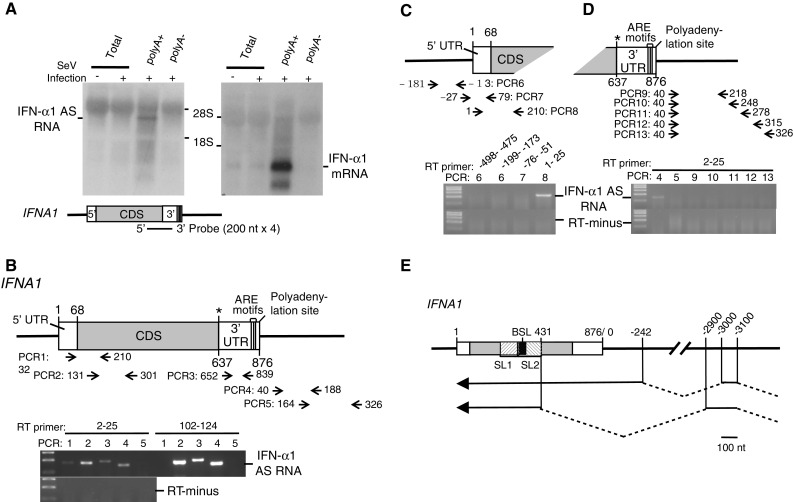
Identification and characterization of IFN-α1 AS RNA. **a** Northern-blot analysis of IFN-α1 AS RNA (*left*) and IFN-α1 mRNA (*right*) in SeV-infected Namalwa cells 24 h after infection. The sense probe employed to identify the AS RNA and the *IFNA1* gene are schematically shown. 28S and 18S show the locations of each rRNA band and were used to estimate the size of IFN-α1 AS RNA. **b** Strand-specific RT was performed for *IFNA1* antisense transcripts with either the 5UF1 RT primer (nt 2–25 of *IFNA1*) or the F1 RT primer (nt 102–124). Both cDNAs were PCR amplified with the primer pairs that span the 5′ UTR and CDS (PCR1) or reside in the CDS (PCR2), 3′UTR (PCR3), or regions downstream of *IFNA1* (PCR4 and PCR5: nt 40–188 and 164–326 from the polyadenylation site of *IFNA1*, respectively). RT-minus indicates a negative PCR control without RT. **c** To localize the 3′ end of IFN-α1 AS RNA, total cellular RNA was analyzed by strand-specific RT-PCR. The RT primers employed were RT1 (nt 1–25 of *IFNA1*), RT-76 (nt −76 to −51 from the transcription initiation site of *IFNA1*), RT-199 (nt −199 to −173) or RT-498 (nt: −498 to −475). PCR6, 7 and 8 depict locations of amplified cDNA fragments relative to the transcription initiation site of *IFNA1*. **d** To localize the 5′ end of IFN-α1 AS RNA, total cellular RNA was analyzed by strand-specific RT-PCR using 5UF1 as the RT primer. PCR9, 10, 11, 12, and 13 depict locations of amplified cDNA fragments relative to the polyadenylation site of *IFNA1*. **e** Genomic location of IFN-α1 AS RNA exons are depicted relative to *IFNA1*. Sequences downstream of the poly(A)^+^ addition site were calculated from nt 876, with nt 876 taken as 0. *Arrows* depict the direction of AS RNA transcription. IFN-α1 AS RNA (*top*) and a splice variant (*bottom*) are shown. *Bar* 10 nt

This result stimulated our interest, since we also recently reported that the NAT of the *inducible nitric oxide synthase* (*iNOS*) gene enhances the stability of iNOS mRNA [18]. Northern-blot analysis using poly(A)^+^ RNA from SeV-infected Namalwa cells (Fig. 2a) revealed that in contrast to the 876 bases IFN-α1 mRNA (DDBJ/EMBL/GenBank Accession Number AB578886), IFN-α1 AS RNA is a ~4-kb RNA, which is transcribed from the positive strand of chromosome 9, which is the opposite strand to the *IFNA1* locus (9p22). We further characterized the naturally occurring IFN-α1 AS RNA by RT-PCR using the antisense strand-specific RT primer, 5UF1 (*IFNA1* nt 2–25) or F1 (nt 102–124). PCR amplification of cDNAs using various primer pairs located across *IFNA1* (Fig. 2b, PCR1-3) and in the downstream region of the gene (Fig. 2b, PCR4,5) revealed the presence of a novel transcript that includes an exon that starts from the downstream region and overlaps the region encoding the entire *IFNA1* (protein coding or “sense”) transcript. The strand specificity of this RT-PCR analysis was supported by the result that PCR1 generated an expected sized product (*IFNA1* nt 32–210) only when the AS RNA was reverse-transcribed by 5UF1 but not by F1 (see Fig. 2b, PCR1).

Failure to generate PCR5 products from both cDNAs led to the idea that the start site of the IFN-α1 AS RNA exon is located in a region between 188 and 326 nucleotides downstream of the poly(A)^+^ addition site of *IFNA1* but on the opposite strand. Further localization of the 5′-end of the IFN-α1 AS RNA exon using various reverse primers spanning the sequences between 326 and 164 confirmed the start site to be 188 nucleotides downstream of the poly(A)^+^ addition site of *IFNA1* (Fig. 2d). The AS RNA exon extended to nucleotide 1 of *IFNA1*, which was supported by PCR 7/8, where PCR8 forward primer (*IFNA1* nt 1–25) but not PCR7 forward primer (nt −27 to −1 from the transcription initiation site of I*FNA1*) amplified IFN-α1 AS cDNA (Fig. 2c) (see also Supplementary Table 1 for primer information).

Using rapid amplification of 5′-cDNA ends, the above AS RNA exon was shown to extend 242 nucleotides downstream of the *IFNA1*poly(A)^+^ addition site. The exon joined a 5′ exon in the opposite orientation to *IFNA1*, which was located some 3 kb away from the poly(A)^+^ site (Fig. 2e). We could not, however, detect antisense 5′ ends, possibly because the AS RNA might form elaborate stem loop structures, resulting in failure to complete the primer extension reaction. We also identified a splice variant for IFN-α1 AS RNA. It is of note that both AS RNAs overlap the CSS region of IFN-α1 mRNA (Fig. 2e). These results thus indicate that the IFN-α1 AS RNA is spliced and has a transcription start site further downstream on chromosome 9.

Analysis of poly(A)^+^ RNA from Namalwa cells by Northern blotting (Fig. 2a), strand-specific RT-PCR (Supplementary Fig. 1a) and 5′-RACE (Fig. 2e) showed that IFN-α1 AS RNA is a polyadenylated transcript that includes a 1.1-kb exon that completely overlaps the IFN-α1 mRNA.

**Fig. 3:**
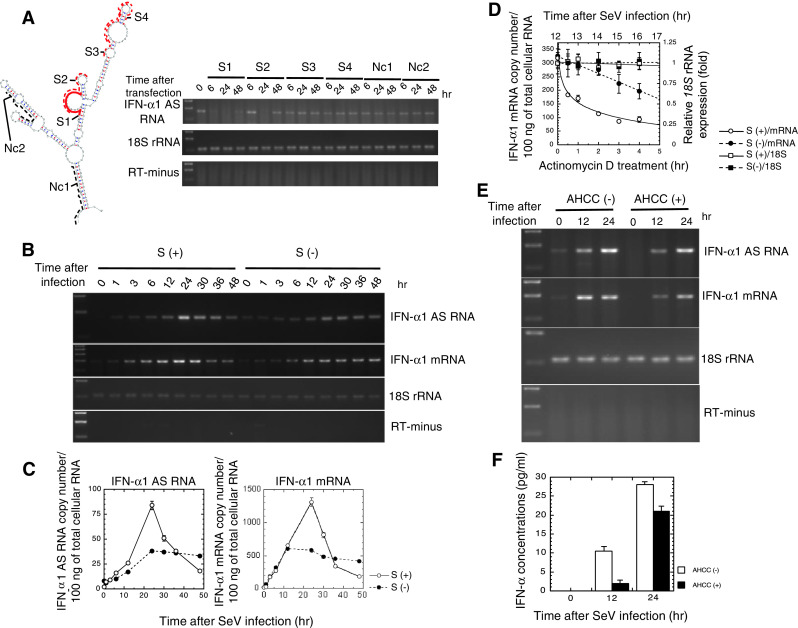
Effect of silencing of IFN-α1 AS RNA on IFN-α1 mRNA expression. **a** Silencing of IFN-α1 AS RNA by sense ODNs. The effect of sense ODNs, S1 (*red bold*), S2-4 (*red dotted*), and Nc1,2 (*black dotted*), on the constitutive expression levels of IFN-α1 AS RNA was examined by strand-specific RT-PCR as described in the legend to Fig. 2b. 18S rRNA indicates an internal RNA standard. **b**, **c** S1- or Nc1-transfected Namalwa cells were subjected to SeV infection as described in the legend to Fig. 2 [**a**, **b** S (+) and S (−), respectively]. RNA samples were collected at the indicated time points after viral infection and subjected to strand-specific RT-PCR (**a**) and real-time PCR (**b**) [S (+)/(−): IFN-α1 AS RNA and IFN-α1 mRNA, respectively]. Copy numbers of IFN-α1 AS RNA and mRNA were determined by the regression lines from a set of standards, as described in the Materials and methods section. The results are presented as the “IFN-α1 AS RNA or mRNA copy number/100 ng of total cellular RNA” ± SEM of triplicate samples. *Open circle* S (+) and *filled circle* S (−) indicate the number of RNA copies in S1- or Nc1-transfected Namalwa cells, respectively. *Error bars* for the real-time analysis of the IFN-α1 AS RNA and mRNA cannot be seen if they are smaller than the graph symbols. **d** The stability of IFN-α1 mRNA over time was measured by real-time PCR relative to time 0 (i.e., 12-h post-infection) after blocking new RNA synthesis with ActD. Copy numbers of IFN-α1 mRNA were quantified and are presented as described in the legend to **c**. The levels of 18S rRNA are presented as “relative 18S rRNA expression” ±SEM of triplicate samples, which indicates the fold change of the 18S rRNA level relative to that at 0 h after ActD treatment in S (−) cells. *Open circle* S (+)/mRNA and *filled circle* S (−)/mRNA indicate the copy numbers of IFN-α1 mRNA in ActD-treated, S1- or Nc1-transfected Namalwa cells, respectively. *Open square* S (+)/18S and *filled square* S (−)/18S indicate the relative 18S rRNA expression levels in ActD-treated, S1- or Nc1-transfected Namalwa cells, respectively. Values from a representative experiment of three independent transfection/infection experiments are shown as the mean ± SEM of triplicate samples. **e**, **f** AHCC reduced IFN-α1 AS RNA levels leading to lower IFN-α1 mRNA/protein levels. The effect of AHCC on IFN-α1 AS RNA and mRNA levels was analyzed by strand-specific RT-PCR. IFN-α secreted by Namalwa cells into the culture supernatant was quantified by ELISA. *Filled square* AHCC (+) and *open square* AHCC (−) indicate IFN-α concentrations obtained in the presence and absence of AHCC, respectively. Values of a representative experiment of three independent infection experiments are shown as the mean ± SEM of triplicate samples

IFN-α1 AS RNA does not contain a long open reading frame, which was to be expected from recent reports from the ENCODE project [26, 27]. A tblastx search of the BLAST database (http://blast.ncbi.nlm.nih.gov/Blast.cgi) with the translated nucleotides of the IFN-α1 AS RNA (DDBJ/EMBL/GenBank AB578885) failed to match any protein sequences (data not shown). We subsequently quantified relative levels of IFN-α1 AS RNA by real-time PCR at the peak 24-h time point after SeV infection (Supplementary Fig. 1b) (see also Fig. 3c). The levels of AS were 4 (Supplementary Fig. 1b) to 6 % (Fig. 3c) of IFN-α1 mRNA.

The *IFNA* multigenic family consists of 13 functional members, and the IFN-α mRNA subtypes are expressed at different levels in Namalwa cells following SeV infection [9]; therefore, the presence of contaminating IFN-α mRNA and IFN-α AS RNA signals other than those from α1 mRNA/AS RNA was examined. A homology search of the RT and PCR primers used to detect IFN-α1 mRNA and AS RNA was performed against other members of the *IFNA* family (Supplementary Table 2). The presence of contaminating IFN-α cDNA signals was subsequently tested using plasmids which contain cDNAs encoding genes with high (*IFNA2*), medium (*IFNA5*) or low (*IFNA17*) homology to *IFNA1*. Each plasmid was digested at either the 5′ or 3′ end and used as a template to identify contaminating sense or antisense RNA signals. The amount of *IFNA1* plasmid was titrated to produce an antisense cDNA signal very close to that obtained from IFN-α1 AS RNA. This amount was then applied to amplifications using *IFNA2*, *A5* and *A17* plasmids. As shown in Supplementary Fig. 2, the F1B/R1 primer pair that was employed to detect IFN-α1 AS RNA did not cross-react with any of the three antisense cDNA mimics examined.

The subsequent cross-contamination assay using the F2B/R2 primer pair to detect IFN-α1 mRNA showed that almost all of the cDNA signals were derived from IFN-α1 mRNA; however 5 % were derived from IFN-α2 mRNA (Supplementary Fig. 2). In contrast, high levels of sequence homology between *IFNA1* and *IFNA13* (99.7 % in the coding region) did not allow distinction between these genes. The expression levels of IFN-α1 mRNA and AS RNA presented in this study thus represent the total amounts of both IFN-α1 and -α13 mRNA/AS RNA.

### Effect of silencing IFN-α1 AS RNA on IFN-α1 mRNA levels

To investigate the possible regulation of IFN-α1 mRNA stability by the NAT of *IFNA1*, we aimed to analyze changes in mRNA levels after silencing IFN-α1 AS RNA expression. Several seODNs (sequences and locations in the CSS are depicted in Fig. 3a) were tested for their abilities to destroy IFN-α1 AS RNA. This silencing strategy is based on the sequence-specific binding of a seODN to the target AS RNA. When the seODN binds IFN-α1 AS RNA and forms a hybrid, the RNA-DNA hybrid leads to the destruction of the AS RNA [18] by RNase H, a cellular nuclease whose substrates include hybrid nucleic acids [28].

As expected from our previous work [11], S1 and S2 (derived from the BSL in the SL2 of CSS) only managed to silence the constitutive expression of IFN-α1 AS RNA (Fig. 3a, 0 h) in human Namalwa B cells. Other seODNs, which were designed from the sequences of either the peripheral SL area of SL2, SL1, or the basal stem region of the CSS, all failed to silence the expression of IFN-α1 AS RNA. Within the BSL, S1 (designed from the only single-stranded region of the BSL) worked more efficiently than S2 to silence the AS RNA (Fig. 3a). The silencing effects by S1/2 were specific, since 18S rRNA (Fig. 3a) and β-actin mRNA (data not shown) levels were unchanged. Thus, we employed 18S rRNA as an internal standard for the subsequent RT-qPCR studies.

Namalwa cells were then transfected with either S1 or Nc1. As expected, S1 effectively reduced IFN-α1 AS RNA expression at time 0 (Fig. 3b, c: compare S (+)/(−), IFN-α1 AS RNA levels at 0 h). However, expression was restored following SeV infection, reaching maximal levels 24 h after infection with approximately 2-fold higher expression in S (+) cells than in S (−) cells. While S (−) cells then maintained AS RNA expression levels, the S (+) cells had a rapid reduction in levels, by 80 % at 48 h after SeV infection. The time-course of IFN-α1 mRNA expression in S (+) and S (−) cells was very similar to that of IFN-α1 AS RNA. While S (−) cells maintained similar mRNA copy numbers after reaching the maximal level at 12 h after viral infection, S (+) cells rapidly reduced the IFN-α1 mRNA level, by 85 % at 48 h after SeV infection. In both S (+) and S (−) cells, the levels of 18S rRNA, the internal standard for RT-PCR reactions, were unchanged (Fig. 3b), indicating that silencing of IFN-α1 AS RNA did not induce general down-regulation of genes.

To investigate whether the reduction of AS RNA expression levels negatively affected the stability of IFN-α1 mRNA, we assessed mRNA stability by blocking new RNA synthesis with ActD and measuring the loss of IFN-α1 mRNA and 18S rRNA in both S (+) and S (−) cells. In S (+) cells, we found that IFN-α1 mRNA had a shorter half-life compared with that in control S (−) cells (0.93 h vs. 5.11 h; Fig. 3d). 18S rRNA, which is a product of RNA polymerase I, was not affected by ActD treatment.

To further confirm the effect of silencing IFN-α1 AS RNA on mRNA levels, we treated Namalwa cells with AHCC. AHCC was previously found to destabilize iNOS mRNA and a number of other cytokine mRNAs by reducing the expression of AS RNAs [29, 30] (Nishizawa et al., manuscript in preparation). As shown in Fig. 3e, AHCC indeed silenced the constitutive expression of IFN-α1 AS RNA in Namalwa cells at time 0. The compound further reduced AS RNA levels and IFN-α1 mRNA levels at both 12 and 24 h after SeV infection, which led to decreased IFN-α protein production (Fig. 3f). These data, together with the ActD results in S (+) cells, imply that inhibition of IFN-α1 AS RNA expression causes the destabilization of IFN-α1 mRNA and, therefore, the reduction of IFN-α protein production.

### Over-expression of IFN-α1 AS increases the stability of IFN-α1 mRNA

**Fig. 4:**
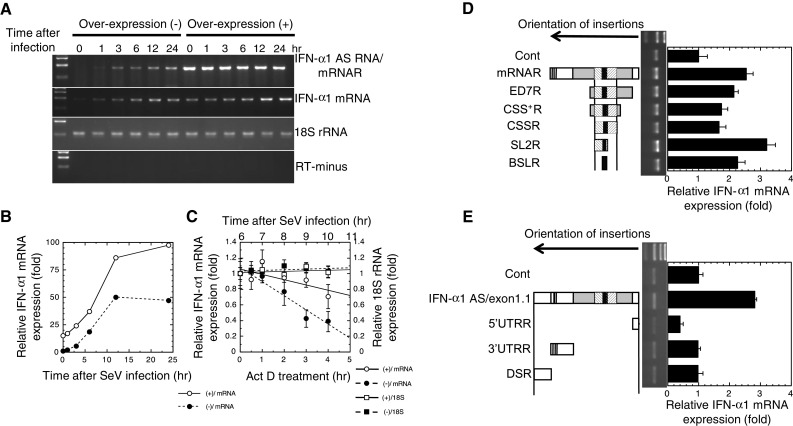
Effect of over-expression of IFN-α1 AS RNA on IFN-α1 mRNA levels. **a** IFN-α1 AS RNA was over-expressed in Namalwa cells by the transfection of the phuIFN-α1/mRNAR expression plasmid. Cells were infected with SeV, and analyzed by strand-specific RT-PCR. **b** The effect of over-expressed IFN-α1 AS RNA on IFN-α1 mRNA levels was quantified by real-time PCR. The levels of IFN-α1 mRNA were normalized to 18S rRNA and are presented as the “relative IFN-α1 mRNA expression” ±SEM of triplicate samples, which indicates the fold change of the mRNA level relative to that at 0 h after SeV infection in mock-transfected cells. *Open circle* (+)/mRNA and *filled circle* (−)/mRNA indicate the relative IFN-α1 mRNA expression levels in the phuIFN-α1/mRNAR- or mock-transfected cells, respectively.* Error bars* cannot be seen when they are smaller than the graph symbols. **c** Over-expressed IFN-α1 mRNAR stabilized IFN-α1 mRNA. The change of stability over time of IFN-α1 mRNA in phuIFN-α1/mRNAR-transfected cells was measured by real-time PCR relative to time 0 (i.e., 6 h after SeV infection) after blocking new RNA synthesis with ActD. The levels of IFN-α1 mRNA were normalized to 18S rRNA and are presented as the “relative IFN-α1 mRNA expression” ±SEM of triplicate samples, which indicates the fold change of the IFN-α1 mRNA level relative to that at 0 h after ActD treatment in mock-transfected cells. The levels of 18S rRNA are presented as described in the legend to Fig. 3d. *Open circle* (+)/mRNA and *filled circle* (−)/mRNA indicate the relative IFN-α1 mRNA expression levels in ActD-treated, phuIFN-α1/mRNAR-, and mock-transfected cells, respectively. *Open square* (+)/18S and *filled square* (−)/18S indicate the relative 18S rRNA expression levels in ActD-treated, phuIFN-α1/mRNAR-, and mock-transfected cells, respectively. **d**, **e** Functional mapping of the stabilization domain of IFN-α1 AS RNA. A series of expression plasmids that express truncated mutant AS RNAs were individually transfected into Namalwa cells. The mutant AS RNAs were mRNAR, ED7R, CSS^+^R, CSSR, SL2R, and BSLR RNAs (**d**), and IFN-α1 AS exon1.1, 5′UTRR, 3′UTRR and DSR RNAs (**e**). *Arrows* show the orientation of each fragment of the *IFNA1* gene and the downstream region (DS) inserted to the expression plasmids. Transfected cells were then infected with SeV, and the effect of over-expression of each IFN-α1 AS RNA fragment on IFN-α1 mRNA levels was analyzed by strand-specific RT-qPCR. The mRNA levels were normalized to 18S rRNA and are presented as the “relative IFN-α1 mRNA expression” ±SEM of triplicate samples, which indicates the fold change of the mRNA level relative to that at 24 h after SeV infection of the control, parental vector-transfected cells (**d**, **e**; Cont). IFN-α1 mRNA bands obtained from the cells that over-expressed each truncated IFN-α1 AS RNA are indicated

The relationship between the mRNA and AS RNA was further examined by transiently over-expressing IFN-α1 AS RNA with the IFN-α1 mRNAR expression plasmid (Fig. 4a). Absolute and relative quantification results of both IFN-α1 mRNA and AS RNA were found to be comparable (compare 24 h after SeV-infection in Fig. 3c with Supplementary Fig. 1b); therefore, we employed the relative quantification method for analysis in the following experiments.

Quantification of IFN-α1 mRNA showed that over-expression of its antisense form increased the mRNA levels 15-fold at time 0 and 2-fold at 24 h after SeV infection compared with empty vector [Fig. 4b, (+)/mRNA and (−)/mRNA]. Over-expression of IFN-α1 mRNAR did not affect 18S rRNA (Fig. 4a) or β-actin mRNA levels (data not shown), indicating that the effect was not a general effect on gene expression. To assess whether the increased levels of IFN-α1 mRNA were due to a change of its stability upon IFN-α1 mRNAR over-expression, the half-life of IFN-α1 mRNA in the over-expression (+) cells was measured by real-time PCR after blocking new RNA synthesis with ActD. As shown in Fig. 4c, IFN-α1 mRNA became more stable when mRNAR was over-expressed *in trans* and resulted in an extended half-life of 8.12 h compared with 2.76 h in the empty vector-transfected (−) cells. In contrast, the half-life of 18S rRNA was not affected by ActD treatment in either the over-expression (+) or the empty vector-transfected (−) cells (Fig. 4c; see also Fig. 3d). Thus, silencing of IFN-α1 AS RNA and over-expression of mRNAR demonstrate that the AS RNA increases the stability of IFN-α1 mRNA.

### Crucial role of the antisense BSLR region for IFN-α1 AS-mediated regulation of IFN-α1 mRNA stability

Sense and antisense transcripts may potentially interact through their complementary single-stranded regions, resulting in double-stranded RNA structures that regulate stability, transport, and/or translation of the sense transcript [31]. To determine the interacting regions of IFN-α1 AS RNA with mRNA, we constructed a series of plasmids that express IFN-α1 AS RNAs with various truncations from both the 5′ and 3′ ends (Fig. 4d, e). Over-expression of IFN-α1 mRNAR gave a level of mRNA 2.5-fold higher than that in control, empty vector-transfected cells (Fig. 4d). Subsequent step-wise truncations of the AS RNA to generate IFN-α1/ED7R, CSS^+^R or CSSR RNA caused a reduction in IFN-α1 mRNA levels. However, slightly higher levels were still maintained compared with the control cells. In contrast, further truncations to create IFN-α1/SL2R RNA resulted in maximal IFN-α1 mRNA levels that were 3.1-fold higher than control cells (Fig. 4d). Interestingly, in the cells that over-expressed BSLR RNA, IFN-α1 mRNA levels were 2.3-fold higher than in control cells. This increase accounts for approximately 70 % of the mRNA increase by the over-expression of IFN-α1/SL2R RNA, suggesting that the BSLR region acts as the core element by conferring a major stabilizing function to the SL2R RNA.

We next examined the effects of over-expressing the IFN-α1 AS RNA exon 1.1 (on the antisense strand, nt 188 from the polyadenylation site of *IFNA1* to 1 of *IFNA1*), the 5′/3′ UTRR or the antisense fragment downstream of the 3′ UTR (DSR) (on the antisense strand, nt 188 to 1 from the polyadenylation site of *IFNA1*). As shown in Fig. 4e, over-expression of the AS RNA exon 1.1 caused 3-fold higher levels of IFN-α1 mRNA, whereas none of the reverted RNAs outside the IFN-α 1 mRNA CDS region positively affected the mRNA expression levels (Fig. 4e). Interestingly, this increase of IFN-α1 mRNA levels was comparable with that obtained by the over-expression of SL2R RNA. Silencing IFN-α1 AS RNA with S1 reduced the mRNA level by 85 % (Fig. 3c) and resulted in a substantially shorter half-life of 0.93 h (Fig. 3d); therefore, these results suggested that the SL2R sequence, containing the core BSLR sequence, plays an important role in the regulation of IFN-α1 mRNA stability by the AS RNA exon 1.1 and hence by the full-length AS RNA.

**Fig. 5:**
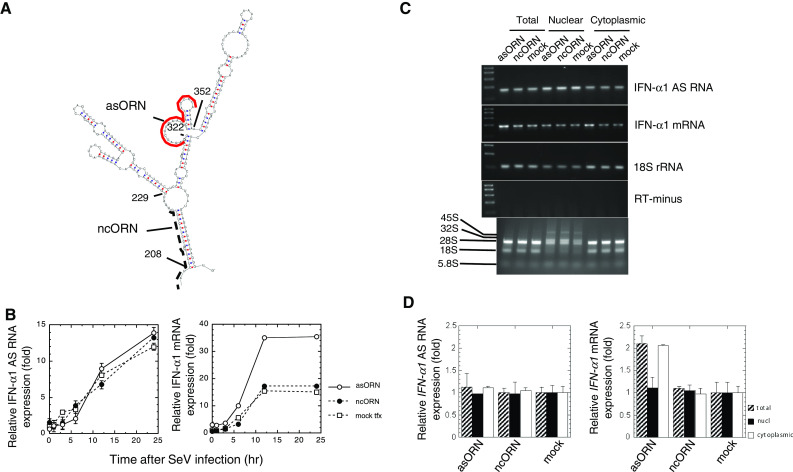
asORN, targeted against the BSL region in the CSS of IFN-α1 mRNA, raised IFN-α1 mRNA expression levels. **a** Location of asORN (*red bold line*) and ncORN (*black broken line*) and the sequences that were taken from the antisense strand of the corresponding regions in the CSS are depicted. **b** To confirm the results obtained by the domain analysis shown in Fig. 4d, either asORN or ncORN was transfected into Namalwa cells. Six hours post-transfection, cells were infected with SeV. RNA samples were then isolated at 1–24 h after viral infection and subjected to strand-specific RT-qPCR. The levels of IFN-α1 AS RNA and IFN-α1 mRNA were normalized to 18S rRNA and are presented as described in the legend to Fig. 4b. *Open circle* ORN, *filled circle* ncORN, and *open square* mock indicate the relative IFN-α1 mRNA expression levels in asORN-, ncORN-, and mock-transfected cells, respectively. *Error bars* cannot be seen when they are smaller than graph symbols. **c**, **d** Total cellular (*filled square*), nuclear (*filled square*) or cytoplasmic (*open square*) RNAs were isolated from asORN-, ncORN-, or mock-transfected cells in **b** but at 24 h after SeV infection. These RNAs were subjected to analysis by strand-specific RT-PCR (**c**) and real-time PCR (**d**). The expression levels of IFN-α1 AS RNA and IFN-α1 mRNA in each RNA fraction were normalized to 18S rRNA and are presented as described in the legend to Fig. 4b, which indicates the fold change of AS RNA or mRNA levels relative to that 24 h after SeV infection in mock-transfected cells. 45S and 32S, as well as 28S, 18S and 5.8S, show positions of nuclear rRNA precursors and rRNAs, respectively

To further confirm the effect of IFN-α1/BSLR RNA on the regulation of mRNA stability, we designed antisense 2′-*O*-methyl oligoribonucleotides (Fig. 5a, asORN) with phosphorothioate bonds [21] to augment IFN-α1 mRNA levels. Such ORNs are small single-stranded RNA fragments that target the BSL of the CSS formed by IFN-α1 mRNA. Both the asORN and ncORN (Fig. 5a) were designed to avoid TLR7/8-mediated IFN-α and TNF-α production by excluding GU-rich motifs [20]. Namalwa cells were either mock transfected or transfected with asORN or ncORN. Six hours after transfection, cells were infected with SeV and subjected to analysis by strand-specific RT-qPCR (Fig. 5b). After transfection of asORN, IFN-α1 mRNA reached 2.3-fold higher levels compared with those expressed in mock-transfected cells. This increase was comparable to that observed using the BSLR RNA expression plasmid. In contrast, ncORN transfection did not change the levels of IFN-α1 mRNA when compared with those observed in mock-transfected cells (Fig. 5b). Neither asORN nor ncORN had any effect on IFN-α1 AS RNA or 18S rRNA expression (Fig. 5c).

The effect of asORN, together with the results obtained by over-expression of BSLR RNA, thus indicated that the pairing of asORN with the target BSL region raises IFN-α1 mRNA expression levels in the same way as IFN-α1 AS RNA exon 1.1 and mRNA duplex formation did. It is further suggested that this RNA–RNA interaction is required to maintain normal levels of IFN-α1 mRNA in cells upon viral infection.

### IFN-α1 AS RNA regulates IFN-α1 mRNA in the cytoplasm

We further investigated the subcellular location of the AS RNA-mediated stabilization of IFN-α1 mRNA. By analyzing cytoplasmic and nuclear fractions separately, we observed that IFN-α1 mRNA was exclusively increased in the cytoplasm, but not in the nucleus after asORN-transfection (Fig. 5c, d), demonstrating that stabilization of IFN-α1 mRNA occurs in the cytoplasm. The increase of IFN-α1 mRNA expression levels was not caused by aberrant RIG-I (retinoic acid-inducible gene-I) recognition of asORN, a chemically synthesized 5′OH ssRNA, or 5′OH dsRNA formed between asORN and the BSL region of IFN-α1 mRNA, because these molecules lack the 5′ triphosphate structure, a hallmark of viral RNAs, which is the prerequisite for RIG-I recognition [32, 33].

In addition, cDNA sequencing of nuclear IFN-α1 mRNA and AS RNA showed that deamination of adenosines to inosines on each strand of the CSS [34] was not observed (sequencing results are deposited in DDBJ/EMBL/GenBank Accession Numbers AB578885 and 6). These results strongly suggest that IFN-α1 AS RNA recognizes mRNA at the BSL of the CSS in the cytoplasm.

### Concordant expression profiles of IFN-α1 mRNA/AS RNA in guinea pig 104C1 fetal fibroblasts infected with PR8 virus

**Fig. 6:**
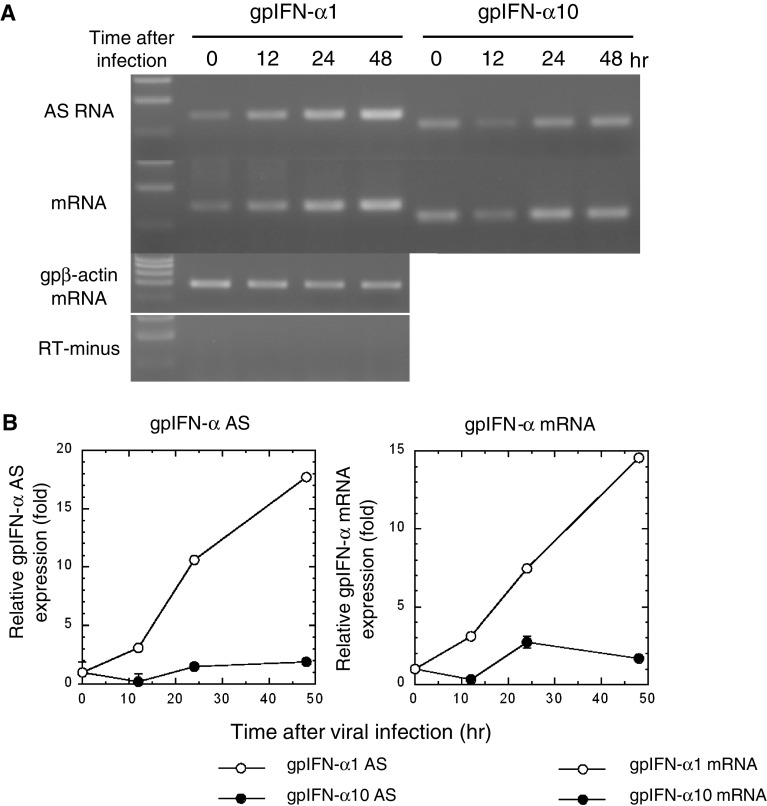
gpIFN-α1 AS RNA and mRNA show concordant expression profiles upon *influenza* A/PR/8/34 (PR8) virus infection in 104 C1 cells. **a**, **b** RNA samples isolated from PR8 virus-infected 104 C1 cells were subjected to strand-specific RT-PCR (**a**) and real-time PCR (**b**) to analyze the expression profile of both gpIFN-α1 AS RNA and IFN-α1 mRNA. Each RNA level was normalized to gpβ-actin mRNA and is presented as the “relative RNA expression” ±SEM of triplicate samples, which indicates the fold change of each RNA level relative to that at 0 h after PR8 virus infection. *Open circle* and *filled circle* indicate the relative expression levels of gpIFN-α1 AS RNA/mRNA and gpIFN-α10 AS RNA/mRNA, respectively. *Error bars* in the real-time analysis of AS RNA/mRNA levels cannot be seen when they are smaller than the graph symbols. gpβ-actin mRNA indicates an internal RNA standard

Strand-specific RT-qPCR analysis of gpIFN-α1 AS RNA indicated that the AS RNA was constitutively expressed in 104C1 cells (Fig. 6a, AS time 0), as was the case in human Namalwa B lymphocytes. Expression levels steadily increased for 48 h after viral infection. GpIFN-α1 mRNA showed a concordant expression profile, with levels steadily increasing for 48 h after PR8 infection (Fig. 6a, b). These expression profiles thus suggested that there is concordant regulation in the expression of gpIFN-α1 AS RNA/mRNA, similar to the observations in SeV-infected Namalwa cells. In contrast, the expression levels of sense/antisense transcripts of gpIFN-α10, which does not encode a functional antiviral protein (Jiang and Kimura, unpublished work; see also [35]), were not changed (Fig. 6a, b). This result indicated that the concordant increase of both gpIFN-α1 mRNA/AS RNA levels was not a general effect in response to viral infection.

### IFN-α1 AS RNA and miR-1270 compete for IFN-α1 mRNA binding

**Fig. 7:**
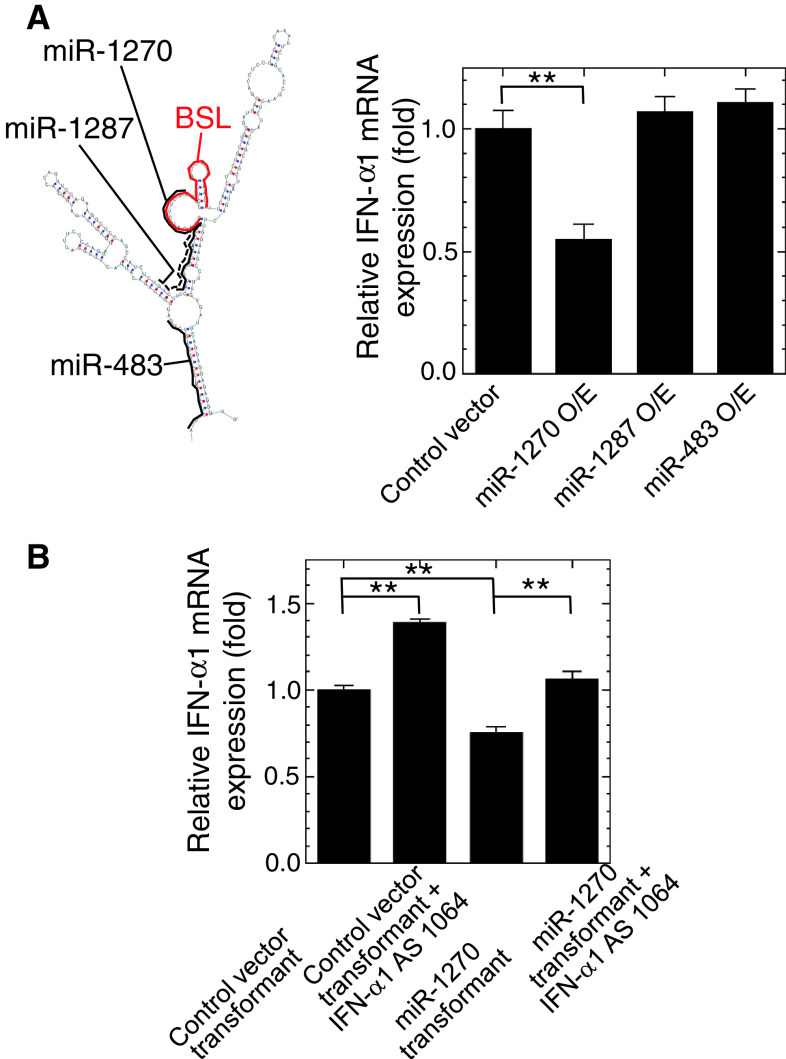
IFN-α1 AS RNA and miR-1270 compete for the same binding site in the BSL region within the CSS of IFN-α1 mRNA. **a** The schematic shows the predicted target sites for miR-1270 (*black bold line*), -1287 (*black dotted line*), and -483 (*black bold line*) and their relation to the BSL region (*red bold line*) of IFN-α1 mRNA. The binding site for miR-1270 is located in the BSL region that is also targeted by IFN-α1 AS RNA. Over-expression of miR-1270, but not of miR-1287 or miR-483, led to IFN-α1 mRNA reduction by about 45 % (**p < 0.01). IFN-α1 mRNA levels were normalized to 18S rRNA and are presented as the “relative IFN-α1 mRNA expression” ±SEM of triplicate samples, which indicates the fold change of the mRNA level relative to that at 24 h after SeV infection of the control, pEGFP-miR-null vector-transfected cells. **b** Over-expression of IFN-α1 AS RNA exon1064 in the recombinant LKO-miR-1270 lentivirus-transduced Namalwa cells can effectively block the reduction of IFN-α1 mRNA expression levels, which was observed in the transduced cells alone. IFN-α1 mRNA levels were normalized to 18S rRNA and are presented as the “relative IFN-α1 mRNA expression” ±SEM of triplicate samples, which indicates the fold change of the mRNA level relative to that at 24 h after SeV infection of the control, LKO-miR-null virus-transduced cells (**p < 0.01)

MiRNAs constitute a class of noncoding regulatory RNAs that functions by binding to target RNAs [36]. We conducted bioinformatic searches for miRNA binding sites in human IFN-α1 mRNA, and predicted the presence of a binding site for miR-1270 in the BSL region (Fig. 7a left). Considering the possible RNA duplex formation between IFN-α1 mRNA and AS RNA in the cytoplasm (Fig. 5c, d), we postulated that a regulatory function of IFN-α1 AS RNA may be “masking” the miR-1270 binding site and thereby blocking the destabilization effects [37] of this miRNA on IFN-α1 mRNA. Other miRNA target sites were also found in the double-stranded “stem” regions adjacent to the single stranded BSL region (Fig. 7a left). We selected two of these miRNAs, including miR-1287, and miR-483, that may share the target site with the Nc1 seODN that failed to silence IFN-α1 AS RNA (Fig. 3a). We over-expressed these miRNAs in SeV-infected Namalwa cells. Exogenous and endogenous expression of these miRNAs were verified by quantification of miR-1270, -1287, and -483 in the transfected and control Namalwa cells (data not shown) (primer information for this assay is shown in Supplementary table 1). We found that miR-1270, which targets the single-stranded BSL region, reduced IFN-α1 mRNA levels, whereas neither miR-1287 nor -483, which target double-stranded stems adjacent to the BSL, were able to alter the mRNA levels (Fig. 7a, right).

To further validate whether IFN-α1 AS RNA and miR-1287 share a potential binding site in the BSL region of IFN-α1 mRNA, we examined the possible counteraction of these ncRNA transcripts. Over-expression of IFN-α1 AS RNA in LKO-null virus-transduced Namalwa cells consistently raised IFN-α1 mRNA levels 24 h after SeV infection. LKO-miR-1270 virus-transduced cells had significantly reduced IFN-α1 mRNA levels, whereas subsequent over-expression of IFN-α1 AS RNA returned the mRNA levels to the basal level (Fig. 7b).

These data thus indicate that miR-1270 and IFN-α1 AS RNA may compete for binding to IFN-α1 mRNA. This proposed miRNA masking effect is compatible with the concordant antisense regulatory action of IFN-α1 AS RNA on the mRNA level.

## Discussion

We previously reported that truncation of the BSL sequence from the CSS, which is located in the 5′ half of the coding region of IFN-α1 mRNA, led to a failure to detect IFN-α1/ED7 ∆BSL mRNA [11]. IFN-α1/ED7 mRNA is the shortest functional IFN-α1 mRNA deletion mutant and the CSS is conserved between the secondary structures of both the mutant and the wild-type mRNAs. These results suggest that the BSL structure may contribute to the regulation of IFN-α1 mRNA stability [11].

In the present study, we present several lines of evidence that this is indeed the case: (1) we identified a natural IFN-α1 AS RNA, a ~4 kb ncRNA that is transcribed from the positive strand of chromosome 9, which is the opposite strand of the *IFNA1* locus (9p22). The IFN-α1 AS RNA encodes an exon that starts 242 nucleotides downstream of the poly(A)^+^ addition site of the gene and overlaps the region encoding the entire *IFNA1* (protein coding or “sense”) transcript. This exon joined a 5′ exon in the opposite orientation to *IFNA1*, which is located some 3 kb away from the poly(A)^+^ site. (2) We showed that S1, which was designed from the BSL sequence, silenced IFN-α1 AS RNA expression and caused destabilization of IFN-α1 mRNA. Treatment of Namalwa cells with AHCC confirmed the silencing effect on IFN-α1 mRNA levels and resulted in the reduction of IFN-α protein expression. (3) Conversely, over-expression of IFN-α1 AS RNA caused the stabilization of the mRNA; i.e., transfection of phuIFN-α1/BSLR or asORN (designed from the antisense strand sequence of the BSL) resulted in increased IFN-α1 mRNA levels. (4) Furthermore, concordant expression profiles of IFN-α1 AS RNA/mRNA were not specific to SeV-infected human Namalwa B lymphocytes but were also observed in PR8 virus-infected guinea pig 104 C1 fibroblasts. These results thus strongly suggest that IFN-α1 AS RNA has a major impact on IFN-α1 mRNA and protein levels by controlling IFN-α1 mRNA expression.

Furthermore, we investigated the subcellular location of asORN-mediated IFN-α1 mRNA stabilization. Cytoplasmic but not nuclear levels of IFN-α1 mRNA were increased upon asORN transfection, demonstrating that stabilization of IFN-α1 mRNA occurs in the cytoplasm independent of the nucleus. This also suggests that IFN-α1 AS RNA and mRNA interact in the cytoplasm, which was further supported by the finding that the deamination of adenosines to inosines on each strand of the nuclear CSS was not observed. Therefore, in the nucleus, which contains an enzyme of the adenosine deaminase family that acts on RNA [34], IFN-α1 AS RNA does not recognize the mRNA to form RNA duplexes. IFN-α1 mRNA and AS RNA interact through their complementary BSL regions, resulting in double-stranded RNA structures in the cytoplasm that regulate mRNA stability.

This RNA duplex formation should, however, be transient in order to avoid recognition by RIG-I [38, 39], which would result in repressed protein synthesis and culminate in apoptosis via protein kinase PKR and 2′-5′-oligoadenylate synthetase (reviewed by [40]).

Quantification of RNA copies revealed that IFN-α1 AS RNA was expressed at an approximately 15-fold lower level than the IFN-α1 mRNA at the peak of expression, 24 h after SeV infection. A number of other studies of mRNA-AS RNA pairs have indicated a similar discrepancy between the levels of the two types of RNAs and yet have found that the AS RNA regulates the mRNA (reviewed by [41]). In fact, a lower expression level of the AS RNA appears to be the rule [26]. A transient interaction between the complementary transcripts has been postulated to allow the AS RNA to move on and target the next mRNA molecule in a “hit-and-run” manner [31, 42]. Therefore, it might be possible to assume that AS RNAs are local modulators of mRNA secondary or tertiary structure [42], the binding of which could result in some type of permanent modification of the IFN-α1 mRNA structure that protects it from degradation even after detachment of IFN-α1 AS RNA. Indeed, the effect of *p15* silencing by *p15* AS persisted after antisense transcription was eliminated by either a tetracycline-inducible vector or by loxP/Cre excision [42]. These proposed mechanisms may explain functionality despite the low abundance of AS RNA molecules (reviewed by [5, 41]).

In light of this, it is interesting to note that among reported *cis*-AS RNAs that have thus far been identified and characterized in human cells ([5, 43] and references therein), double-stranded RNAs have proved difficult to identify in most cases [44], suggesting that such endogenous sense/antisense duplex formation may be too transient and/or labile to allow detection [18, 31].

Recently, Wahlestedt and coworkers [45] presented evidence to show that the miR-485-5p transcript and an antisense β-site amyloid precursor protein-cleaving enzyme 1 (BACE1) transcript (BACE1-AS) compete for a binding site on BACE1 mRNA. Considering the reported effects of miRNAs on mRNA stability ([46], see also [37]), they proposed a molecular mechanism whereby cytoplasmic sense-antisense RNA duplex formation can potentially inhibit the interaction between miR-485-5p and BACE1 mRNA to explain the enhancement of BACE1 mRNA stability by BACE1-AS transcripts.

We subsequently searched for miRNA recognition motifs in the entire IFN-α1 mRNA sequence, and in the CSS sequence in particular, and found that a recognition site for miR-1270 could be reliably predicted (RNAhybrid in the BiBiserv: http://bibiserv.techfak.uni-bielefeld.de/rnahybrid/; [47], and RegRNA: http://RegRNA.mbc.NCTU.edu.tw/; [48]). MiR-1270 recognizes nt 308–333 in the CSS and has the highest assigned score and lowest predicted ∆G of binding with IFN-α1 mRNA. The fact that the BSL spans nt 322–352 and that the asORN was designed from nt 322–345 in the CSS of IFN-α1 mRNA suggests that the transient cytoplasmic sense-antisense RNA duplex formation discussed above alters the secondary or tertiary structure of IFN-α1 mRNA (the BSL RNA region in particular), which blocks the miRNA-recognition site owing to its overlapping with the single-stranded region within the BSL.

Indeed, destabilization of IFN-α1 mRNA caused by miR-1270 was counteracted by IFN-α1 AS RNA, leading to the full recovery of IFN-α1 mRNA expression to the basal level. Hence, the proposed action of IFN-α1 AS RNA, promoting target mRNA stability by duplex formation and inhibiting mRNA destabilization by miRNA, serve to elevate IFN-α1 mRNA and protein levels upon viral infection.

Our data not only identify a mechanism for post-transcriptional *IFNA1* gene regulation but also highlight the role of human antisense transcripts as regulatory RNAs [5, 49]. Growing evidence suggests that antisense transcription has a key role in a range of human diseases (reviewed by [50]). Activation of the immune system causes the rapid production of IFNs and pro-inflammatory cytokines that orchestrate the developing innate and adaptive immune responses to infection. Aberrant or excessive stimulation of these pathways is believed to underlie many inflammatory autoimmune disorders. For example, RNA- and DNA-associated autoantigens in systemic lupus erythematosus have been shown to drive pathological expression of type I IFN genes and IFN-induced genes through TLR7 and TLR9 activation (reviewed by [51] and see references therein). Our discovery of IFN-α1 AS RNA as a regulator of the *IFNA1* gene at the mRNA level raises the possibility that dysfunction of the AS RNA could contribute to autoimmunity by failure to sustain *IFNA1* gene expression and function. Thus, investigation into the status of IFN-α1 AS RNA expression in autoimmune disorders is highly warranted.

In conclusion, IFN-α1 AS RNA adds another level of complexity to the regulation of *IFNA1*. Further studies of AS RNA may provide a more complete understanding of the regulation of *IFNA1* in various physiological processes, as well as during autoimmune disease development, and may lead to therapeutic intervention.

### Electronic supplementary material

Below is the link to the electronic supplementary material.

Supplementary material 1 (PDF 151 kb)

**Supplementary Fig. 1** Characterisation and quantification of IFN-α1 AS RNA. **a** To further characterise the AS RNA, total cellular, poly(A)^+^ and poly(A)^-^ RNAs were extracted and prepared from SeV-infected Namalwa cells 24 h after infection and were analysed by strand-specific RT-PCR using the F1 primer and the F1B/R1 primer pair as described in Materials and methods. 28S and 18S show positions of rRNA bands. RT-minus indicates a negative PCR control without RT. **b** Quantification of IFN-α1 AS RNA by strand-specific RT-qPCR. Total cellular RNA was prepared from the cells described above. Strand-specific RT was performed for IFN-α1 mRNA (mRNA) and its AS RNA (AS), followed by real-time PCR with the respective primer pairs (□ F1B/R1 or ■ F2B/R2). Values from a representative experiment of three independent infection experiments are shown as the “relative RNA level” ± s.e.m. of triplicate samples, where the level of IFN-α1 mRNA is denoted as 100%. (PDF 73 kb)

**Supplementary Fig. 2** Evaluation of the specificity of the real-time PCR assay for the quantification of IFN-α1 AS RNA/mRNA signals. To evaluate the presence of contaminating IFN-α cDNA signals, the F1B/R1 and F2B/R2 primer pairs were tested for amplification of *IFNA1, 2, 5 and 17* or their revertants. The amount of *IFNA1* plasmid was titrated to produce an antisense cDNA signal very close to that obtained from IFN-α1 AS RNA. This amount was then applied to amplifications using *IFNA2*, *A5* and *A17* plasmids. Values from a representative experiment of three independent experiments are shown as the “relative expression level” ± s.e.m. of triplicate samples, where the levels of IFN-α1 AS RNA and IFN-α1 mRNA are denoted as 100%. (PDF 71 kb)

## Abbreviations

lncRNA: Long non-coding RNA
NAT: Natural antisense transcript
IFN-α1: Interferon-α1
*IFNA*: *IFN*-*α* gene
AS RNA: Antisense RNA
miR: microRNA
SeV: *Sendai virus*
PR8 virus: *Influenza virus* A/PR/8/34
CSS: Conserved secondary structure
SL: Stem loop
BSL: Bulged-stem loop
UTR: Untranslated region
seODN: Sense oligodeoxynucleotide
asORN: Antisense oligoribonucleotide
AHCC: Active hexose correlated compound
TLR: Toll-like receptor
RACE: Rapid amplification of cDNA ends
iNOS: Inducible nitric oxide synthase
ActD: Actinomycin D
RT-qPCR: Reverse-transcription quantitative polymerase chain reaction
Gp: Guinea pig
R: Revertant
IFN-α1 mRNAR: Revertant form of IFN-α1 mRNA
DSR: Antisense fragment downstream of *IFNA1* 3′ UTR
BACE1: β-site amyloid precursor protein-cleaving enzyme 1
miRNA: microRNA
