# Supplementary material for: Stabilization of human interferon-α1 mRNA by its antisense RNA
Source: Cell Mol Life Sci. 2012 Dec 8;70(8):1451–67. doi: 10.1007/s00018-012-1216-x (PMC3607724; doi:10.1007/s00018-012-1216-x)
Supplement: Supplementary file 1 — Supplementary material 1 (PDF 151 kb) [file 18_2012_1216_MOESM1_ESM.pdf]

Supplementary Table 1. List of primers used in this study.

IFN-α superfamily cross reactivity assay[a]

For construction of IFN-α2 expression vector[a]

α2F, 5'-cccaagcttGAGAACCTGGAGCCTAAGGTTTAG-3' (*IFNA2* nt 1-24)

α2R, 5'-tgctctagaTGGTTTACAGAAAGTTTATTTTTAC-3' (*IFNA2* nt 1143-1119)

For construction of IFN- α5 expression vector

α5flankF, 5'-CTGAGAAACACTCCTGTACATCTATGTAG-3' (nt -146- -118 from the transcription initiation site of *IFNA5*)

α5flankR, 5'-GTCCCTGTGGAGCTAAAGAAGTG-3' (nt 95-73 in the antisense strand from the polyadenylation site of *IFNA5*)

α5F, 5'-cccaagcttGCCCAAGGTTCAGGGTCAC-3' (*IFNA5* nt 1-19)

α5R, 5'-gctctagaTGAAACGGCAGAACTCAAGAAGTGTG-3' (*IFNA5* nt 700-675)

For construction of IFN-α17 expression vector

α17flankF, 5'-AAAAACAATGAAAAACATTCTTAAACAC-3' (nt -162- -135 from the transcription initiation site of *IFNA17*)

α17flankR, 5'-GTCAGCCTCGACATTTTATCTTAAA-3' (nt 49-25 in the antisense strand from the polyadenylation site of *IFNA17*)

α17F, 5'-cccaagcttGTTCAAGGTTACCCATCTCAA-3' (*IFNA17* nt 1-21)

α17R, 5'-gctctagaGTATAGTAAAAATTTAATGAAAAAGGAAATTA-3' (*IFNA17* nt 980-949)

For construction of northern blotting probes

IFNA1-AS 301-320 *Bam*HI-F, 5'-cgcggatccTGTCAGAGCAGAAATCATGA-3' (IFNA1-AS nt 301-320)

IFNA1-AS 500-468 *Bgl*II R, 5'-gaagatctTAGATAAATCAGTTTATCAGCATGGTCATAGT-3' (IFN-AS nt 500-468)

Primers employed for 5'-RACE analysis

Biotinylated F1, B-TGGTGCTCAGCTGCAAGTCAAGC-3' (*IFNA1* nt 101-124)

CA cassette adaptor (Takara Bio), 5'-GCGTGGTACCATGGTCTAGAGTCGACTAAGTAGGT-3'

Forward primers F1B, 5'-CTGGGCTGTGATCTCCCTGAGAC-3' (*IFNA1* nt 131-153)

F2, 5'-CTGATCCAGCAGATCTTCAAC-3' (*IFNA1* nt 314-334)

Reverse primer CA primer, (Takara Bio) 5'-CGTGGTACCATGGTCTAGAGT-3'

For construction of IFN-α1 AS fragment expression vectors

IFN-α1 AS

IFN-α1 AS/F, 5'-cccaagcttAGAACCTAGAGCCCAAGGTTCAG-3' (*IFNA1* nt 1-23)

IFN-α1 AS/R, 5'-gctctagaATACAACCTGGTTTAGAGAAAGGTCA-3' (nt 188-164 in the antisense strand from the polyadenylation site of *IFNA1*)

ED7R

ED7/F, 5'-gctctagaATGGCCTCGCCCTTTGCTTTACTG-3'1 (*IFNA1* nt 68-91)

ED7/R, 5'-cgtctagaGATGGAGTCCGCATTCATCAGGG-3' (*IFNA1* nt 487-465)

CSS^+R

CSS^+/F, 5'-cccaagcttATGCTCCTGGCACAAATGAGCAG-3'(*IFNA1* nt 182-204)

CSS$^+$/R, 5'-gctctagaAGATGGAGTCCGCATTCATCAG -3' (*IFNA1* nt 488-467)

CSSR

CSS/F, 5'-cccaagcttCTCTCCTTCCTCCTGTCTGATG-3' (*IFNA1* nt 208-229)

CSS/R, 5'-gctctagaCCCTCTCCTCCTGCATCACAC-3' (*IFNA1* nt 452-432)

SL2R

SL2/F, 5'-cccaagcttCATGAGCTGATCCAGCAGATC-3' (*IFNA1* nt 308-328)

SL2/R, 5'-gctctagaCACAGGCTTCCAAGTCATTCAG-3' (*IFNA1* nt 434-413)

BSLR

BSL/F, 5'-agcttGCAGATCTTCAACCTCTTTACCACAAAAGATt-3' (*IFNA1* nt 322-352)

BSL/R, 5'-ctagaATCTTTTGTGGTAAAGAGGTTGAAGATCTGCa-3' (*IFNA1* nt 352-322)

5'UTRR

IFN-α1 AS/F, as above

5'UTR/R, 5'-gctctagaCGTAGATATTGCAGATACTTCTGGGC-3' (*IFNA1* nt 67-42)

3'UTRR

3'UTR/F, 5'-cccaagcttCATCTGGTCCAACATGAAAACAATTC-3' (*IFNA1* nt 638-663)

3'UTR/R, 5'-gctctagaGAGTAAATATAAGGAACATGTTTTATTACACTAAC-3' (*IFNA1* nt 876-842)

DSR

DSR/F, 5'-cccaagcttAATCCATTATTTTGTGTTGTTCATTAAACTTTTACTATAG-3' (nt 1-41 from the polyadenylation site of *IFNA1*)

DSR/R, 5'-gctctagaATACAACCTGGTTTAGAGAAAGGTC-3' (nt 188-140 in the antisense strand from the polyadenylation site of *IFNA1*)

For construction of miRNA expression vectors

pEGFP-miR-1270

Flank-miR-1270F, 5'-tcgaggatccTGAACTCCTGACCTCAAGTGATTCACCC-3' (*Homo sapiens* chromosome 19, GRCh37.p2 primary reference assembly AS 339972-339999)

Flank-miR-1270R, 5'-tcgagctagcTCCTGGACATATAAATGTAACAAAAACATACTGACC-3' (*Homo sapiens* chromosome 19, GRCh37.p2 primary reference assembly 20579140 -20579105)

pEGFP-miR-1287

Flank-miR-1287F, 5'-tcgaggatccCAGCTCCCATTACCAAGGTGAGTGGTTC-3' (*Homo sapiens* chromosome 10, GRCh37.p2 primary reference assembly AS 49856-49883)

Flank-miR-1287R, 5'-tcgagctagcTGCCACTGCCACTGTTGTCCTCACTCC-3' (*Homo sapiens* chromosome 10, GRCh37. p2 primary reference assembly 100154875-100154849)

pEGFP-miR-483

Flank-miR-483F, 5'-tcgaggatccGCTCCTGCTCCCTCCTCCGGCTG-3' (*Homo sapiens* chromosome 11 genomic contig, GRCh37.p2 primary reference assembly 2095539-2095517)

Flank-miR-483R 5'-tcgagctagcGACTGCTGTCCCTGAGCTTGGACTCTGGC-3' (Homo sapiens chromosome 11 genomic contig, GRCh37. p2 primary reference assembly 2095264-2095292)

For detection of pri-/pre-miR-1270, -1287 and -483

Pri-/pre-miR-1270

Pri/pre-miR-1270F, 5'-CACAGAGTTATACTGGAGATATGGAAGAGC-3' (MIR1270-1 nt 1-30)

Pri/pre-miR-1270R, 5'-TGCAAAGAGCCACATAGAAGATAAAGAAAAGCC-3' (MIR1270-1 nt 83-51)

Pri-/pre-miR-1287

Pri/pre-miR-1287F, 5'-GTTGTGCTGTCCAGGTGCTGGATC-3' (MIR1287 nt 1-24)

Pri/pre-miR-1287R, 5'-GTCATGGCTCCAATCACTGCATCTGTG-3' (MIR1287 nt 88-62)

Pri-/pre-miR-483

Pri/pre-miR-483F, 5'-GAGGGGGAAGACGGGAGGAAAG-3' (MIR483 nt 1-22)

Pri/pre-miR-483R, 5'-GAGAGGAGAAGACGGGAGGAGAG-3' (MIR483 nt 76-53)

Strand-specific RT-qPCR

For detection of IFN-α1 mRNA

RT primer: R2, 5'-GGATCTCATGATTTCTGCTCTGAC-3' (*IFNA1* nt 588-566)

PCR primer pair: F2B, 5'-CTCTACCAGCAGCTGAATGACTT-3' (*IFNA1* nt 401-423) /R2

For detection of IFN-α1 AS

RT primer: F1, 5'-TGGTGCTCAGCTGCAAGTCAAGC-3' (*IFNA1* nt 102-124)

PCR primer pair: F1B, 5'-CTGGGCTGTGATCTCCCTGAGAC-3' (*IFNA1* nt 131-153) /R1, 5'-AGAGATGGCTGGAGCCTTCTG-3' (*IFNA1* nt 301-281)

For detection of 18S rRNA

PCR primer pair: *18S* F, 5'-CTTAGAGGGACAAGTGGCG-3' (human 18S rRNA nt 1443-1461)/*18S* R, 5'-ACGCTGAGCCAGTCAGTGTA-3' (human 18S rRNA nt 1549-1529)

For detection of gpIFN-α1 mRNA

RT primer: gpR1, 5'-CACAGTGTTGCCCAGTACTCCTGA-3' (Guinea pig IFN-α1 cDNA nt 710-687)

PCR primer pair: gpF1B, 5'-AGAATGAGTCTGGGTTTAACACGGAA-3' (Guinea pig IFN-α1 nt 565-590)/ gpR1

For detection of gpIFN-α1 AS

RT primer: gpF1, 5'-GCCTGAAGCACAGACAGGACTTTG-3' (Guinea pig IFN-α1 cDNA nt 155-178)

PCR primer pair: gpF1B/gpR1

For detection of gpβ-actin mRNA

gpβ-actinF, 5'-CTGTCTGGTGGTACCACCATG-3' (gpβ-actin cDNA nt902-922)

gpβ-actinR, 5'-CTCCTGCTTGC GATCCAC-3' (gpβ-actin cDNA nt 1090-1072)

Characterisation of IFN-α1 AS

For localisation of its 5' end

RT primers: 5UF1, 5'-GAACCTAGAGCCCAAGGTTCAGAG-3' (*IFNA1* nt 2-25); F1, as above

PCR primer pairs:

PCR1F, 5'-ATCTCAGCAAGCCCAGAAGTATC-3'(*IFNA1* nt: 32-54)/PCR1R, 5'-GAGATTCTGCTCATTTGTGCCAG-3' (*IFNA1* nt: 210-188),

F1B/R1 (as above for PCR2),

PCR3F, 5'-TGAAAACAATTCTTATTGACTCATAC-3' (*IFNA1* nt 652-677)/PCR3R, 5'-CAGTGTAAAGGTGCACATGACG-3' (*IFNA1* nt 839-818),

PCR4F, 5'-GGAACTTCCTGTATGTGTTCATTC-3' (nt 40-63 from the polyadenylation site of

IFNA1)/PCR4R, 5'-ATACAACCTGGTTTAGAGAAAGGTC-3' (nt 188-164 in the antisense strand from the polyadenylation site of *IFNA1*),

PCR5F, 5'-GACCTTTCTCTAAACCAGGTTGTAT-3' (nt 164-188 from the polyadenylation site of *IFNA1*)/PCR5R, 5'-GCTCCTTCTTCTCATAGTATTTAGG-3' (nt 326-302 in the antisense strand from the polyadenylation site of *IFNA1*),

PCR4F/PCR9R, 5'-TTGTATTCAGCTTTATATCTTGAGTACAAC-3' (nt 218-189 in the antisense strand from the polyadenylation site of *IFNA1*),

PCR4F/PCR10R, 5'-AAATATGCAAGAGAGTAGGAAGTAGATTTA-3' (nt 248-219 in the antisense strand from the polyadenylation site of *IFNA1*),

PCR4F/PCR11R, 5'-CCATCTTTAGTTTTATTCCATACAAAAACC-3' (nt 278-249 in the antisense strand from the polyadenylation site of *IFNA1*),

PCR4F/PCR12R, 5'-TCATAGTATTTAGGATAACTGATATTAAGTATGATTA-3' (nt 315-279 in the antisense strand from the polyadenylation site of *IFNA1*),

PCR4F/PCR13R, 5'-GCTCCTTCTTCTCATAGTATTTAGG-3' (nt 326-302 in the antisense strand from the polyadenylation site of I*FNA1*)

For localisation of its 3' end

RT primer: RT1, 5'-AGAACCTAGAGCCCAAGGTTCAGAG-3' (*IFNA1* nt 1-25), PCR primer pair: RT1/PCR1R (as above for PCR8)

RT primer: RT-76, 5'-GTGGCCCAGAAGCATTAAGAAAGTGG-3' (nt -76- -51 from the transcription initiation site of *IFNA1*)

PCR primer pair: PCR7F, 5'-GGCATTTGCAGGAAGCAAGGCCTTCAG-3' (nt: -27- -1 from the transcription initiation site of *IFNA1*)/PCR7R, 5'-GGGCGAGGCCATCGTAGATATTGCAG-3' (*IFNA1* nt 79-54)

RT primers: RT-199, 5'-GATATCTAAAAAGTCTCTGGGAACAAG-3' (nt -199- -173 from the transcription initiation site of *IFNA1*);

RT-498, 5'-CCCAAAGTGCTGGGATTACAGGCG-3' (nt -498- -475 from the transcription initiation site of *IFNA1*)

PCR primer pair: PCR6F, 5'-GGGAACAAGATGGGGAAGACAATAATG-3' (nt -181- -156 from the transcription initiation site of *IFNA1*)/PCR6R,

5'-CTTCCTGCAAATGCCTTAAATAGGGAAC-3' (nt -13- -40 from the transcription initiation site of *IFNA1*)

Assays for silencing IFN-α1 AS RNA[b]

S1	5'- C*C*A*GCAGATCTTCAACC*T*C*T-3'2 (*IFNA1* nt 319-338)
S2	5'- A*T*C*TTCAACCTCTTTAC*C*A*C-3' (*IFNA1* nt 326-345)
S3	5'- G*A*T*GAGGACCTCCTAGA*C*A*A-3' (*IFNA1* nt 368-387)
S4	5'- G*A*C*CTCCTAGACAAATT*C*T*G-3' (*IFNA1* nt 374-393)
Nc1	5'- A*A*T*CTCTCCTTCCTCCT*G*T*C-3' (*IFNA1* nt 205-224)
Nc2	5'-C*C*A*GGAGGAGTTTGATG*G*C*A-3' (*IFNA1* nt 253-272)

[a]The flanking restriction sites are in lower case type.

[b]An asterisk indicates a phosphorothioate bond.

Supplementary Table 2. Homology searches of primers used to detect IFN-α1 mRNA/AS[a] against *IFNA* gene subtypes.

RT primer: F1, 5'-TGGTGCTCAGCTGCAAGTCAAGC-3' to detect IFN-α1 AS

Gene symbol	Homology	Sequence (Location in the gene)
IFNA1	23/23 (100%)	5'-TGGTGCTCAGCTGCAAGTCAAGC-3' (102-124)
IFNA13	23/23 (100%)	5'-TGGTGCTCAGCTGCAAGTCAAGC-3' (104-126)
IFNA2	23/23 (100%)	5'-TGGTGCTCAGCTGCAAGTCAAGC-3' (103-125)
IFNA6	23/23 (100%)	5'-TGGTGCTCAGCTGCAAGTCAAGC-3' (35-57)
IFNA14	23/23 (100%)	5'-TGGTGCTCAGCTGCAAGTCAAGC-3' (79-101)
IFNA5	21/23 (91%)	5'-TGGTGCTCAACTGCAAGTCAATC-3' (92-114)
IFNA8	20/23 (87%)	5'-TGGTGCTCAGCTACAAGTCATTC-3' (65-87)
IFNA4	19/23 (82%)	5'-TGGTGCTCAGCTACAAATCCATC-3' (103-125)
IFNA10	19/23 (82%)	5'-TGGTGCTCAGCTACAAATCCATC-3' (81-103)
IFNA16	19/23 (82%)	5'-TGGTGCTCAGCTACAAATCCATC-3' (41-63)
IFNA17	19/23 (82%)	5'-TGGTGCTCAGCTACAAATCCATC-3' (84 -106)
IFNA21	19/23 (82%)	5'-TGGTGCTCAGCTACAAATCCATC-3' (83-105)
IFNA7	18/23 (78%)	5'-TGGTACTCAGCTACAAATCCATC-3' (75-97)

Forward PCR primer: F1B, 5'-CTGGGCTGTGATCTCCCTGAGAC-3' to detect IFN-α1 AS

Gene symbol	Homology	Sequence (Location in the gene)
IFNA1	23/23 (100%)	5'-CTGGGCTGTGATCTCCCTGAGAC-3' (131-153)
IFNA13	23/23 (100%)	5'-CTGGGCTGTGATCTCCCTGAGAC-3' (133-155)
IFNA4	21/23 (91%)	5'-CTGGGCTGTGATCTGCCTCAGAC-3' (132-154)
IFNA5	21/23 (91%)	5'-CTGGGCTGTGATCTGCCTCAGAC-3' (121-143)
IFNA7	21/23 (91%)	5'-CTGGGCTGTGATCTGCCTCAGAC-3' (104-126)
IFNA8	21/23 (91%)	5'-CTGGGCTGTGATCTGCCTCAGAC-3' (94-116)
IFNA10	21/23 (91%)	5'-CTGGGCTGTGATCTGCCTCAGAC-3' (110-132)
IFNA16	21/23 (91%)	5'-CTGGGCTGTGATCTGCCTCAGAC-3' (70-92)
IFNA21	21/23 (91%)	5'-CTGGGCTGTGATCTGCCTCAGAC-3' (112-134)
IFNA6	20/23 (86%)	5'-CTGGACTGTGATCTGCCTCAGAC-3' (64-86)
IFNA17	20/23 (86%)	5'-CTAGGCTGTGATCTGCCTCAGAC-3' (113-135)
IFNA2	19/23(83%)	5'-GTGGGCTGTGATCTGCCTCAAAC-3' (132-154)
IFNA14	18/23 (78%)	5'-CTGGGCTGTAATCTGTCTCAAAC-3' (108-130)

Reverse PCR primer: R1, 5'-AGAGATGGCTGGAGCCTTCTG-3' to detect IFN-α1 AS

Gene symbol	Homology	Sequence (Location in the gene)
IFNA1	21/21 (100%)	5'-AGAGATGGCTGGAGCCTTCTG -3' (301-281)
IFNA13	21/21 (100%)	5'-AGAGATGGCTGGAGCCTTCTG-3' (303-283)
IFNA4	20/21 (95%)	5'-AGAGATGGCTTGAGCCTTCTG -3' (302-282)
IFNA5	20/21 (95%)	5'-AGAGATGGCTTGAGCCTTCTG-3' (291-271)
IFNA8	20/21 (95%)	5'-AGAGATGGCTTGAGCCTTCTG-3' (264-244)
IFNA10	20/21 (95%)	5'-AGAGATGGCTTGAGCCTTCTG-3' (280-260)
IFNA16	20/21 (95%)	5'-AGAGATGGCTTGAGCCTTCTG-3' (240-220)
IFNA21	20/21 (95%)	5'-AGAGATGGCTTGAGCCTTCTG -3' (282-262)

IFNA6	19/21 (90%)	5'-AGAGATGGCTTCAGCCTTCTG-3' (234-214)
IFNA7	19/21 (90%)	5'-AGAGATGGCTTGAGTCTTCTG-3' (274-254)
IFNA14	19/21 (90%)	5'-AGAGATGGCTTGAGCTTTCTG-3' (278-258)
IFNA17	19/21 (90%)	5'-AGAGATGGCTTGAGTCTTCTG-3' (283-263)
IFNA2	16/21 (76%)	5'-AGGGATGGTTTCAGCCTTTTG-3' (299-279)

RT primer: R2, 5'-GATCTCATGATTTCTGCTCTGAC-3' to detect of IFN-α1 mRNA

Gene symbol	Homology	Sequence (Location in the gene)
IFNA1	23/23 (100%)	5'-GATCTCATGATTTCTGCTCTGAC-3' (588-566)
IFNA2	23/23 (100%)	5'-GATCTCATGATTTCTGCTCTGAC-3' (586-564)
IFNA4	23/23 (100%)	5'-GATCTCATGATTTCTGCTCTGAC-3' (589-567)
IFNA5	23/23 (100%)	5'-GATCTCATGATTTCTGCTCTGAC-3' (578-556)
IFNA6	23/23 (100%)	5'-GATCTCATGATTTCTGCTCTGAC-3' (521-499)
IFNA7	23/23 (100%)	5'-GATCTCATGATTTCTGCTCTGAC-3' (561-539)
IFNA8	23/23 (100%)	5'-GATCTCATGATTTCTGCTCTGAC-3' (551-529)
IFNA10	23/23 (100%)	5'-GATCTCATGATTTCTGCTCTGAC-3' (567-545)
IFNA13	23/23 (100%)	5'-GATCTCATGATTTCTGCTCTGAC-3' (590-568)
IFNA14	23/23 (100%)	5'-GATCTCATGATTTCTGCTCTGAC-3' (565-543)
IFNA16	23/23 (100%)	5'-GATCTCATGATTTCTGCTCTGAC-3' (527-505)
IFNA17	23/23 (100%)	5'-GATCTCATGATTTCTGCTCTGAC-3' (560-548)
IFNA21	23/23 (100%)	5'-GATCTCATGATTTCTGCTCTGAC-3' (569-547)

Forward PCR primer: F2B, 5'-CTCTACCAGCAGCTGAATGACTT-3' to detect IFN-α1 mRNA

Gene symbol	Homology	Sequence (Location in the gene)
IFNA1	23/23 (100%)	5'-CTCTACCAGCAGCTGAATGACTT-3' (401-423)
IFNA13	23/23 (100%)	5'-CTCTACCAGCAGCTGAATGACTT-3' (403-425)
IFNA2	22/23 (95%)	5'-CTCTACCAGCAGCTGAATGACCT-3' (399-421)
IFNA5	21/23 (91%)	5'-CTTTACCAGCAGCTGAATGACCT-3' (391-413)
IFNA6	21/23 (91%)	5'-CTTTACCAGCAGCTGAATGACCT-3' (334-356)
IFNA4	20/23 (86%)	5'-CTTTACCAGCAACTGAATGACCT-3' (402-424)
IFNA7	20/23 (86%)	5'-CTTTACCAGCAACTGAATGACCT-3' (374-396)
IFNA8	20/23 (86%)	5'-CTTGACCAGCAGCTGAATGACCT-3' (364-386)
IFNA10	20/23 (86%)	5'-CTTTACCAGCAACTGAATGACCT-3' (380-402)
IFNA21	20/23 (86%)	5'-CTTAACCAGCAGCTGAATGACCT-3' (382-404)
IFNA16	19/23 (82%)	5'-CTTTTCCAGCAACTGAATGACCT-3' (340-362)
IFNA17	19/23 (82%)	5'-CTTTACCAGCAACTGAATAACCT-3' (383-405)
IFNA14	18/23 (78%)	5'-CTTTTCCAGCAAATGAATGACCT-3' (378-400)

Reverse PCR primer: R2, 5'-GATCTCATGATTTCTGCTCTGAC to detect of IFN-α1 mRNA

Gene symbol	Homology	Sequence (Location in the gene)
See above		

[a]Red characters denote mismatched sequences.
