# Supplementary figures and images for: Stabilization of human interferon-α1 mRNA by its antisense RNA
Source: Cell Mol Life Sci. 2012 Dec 8;70(8):1451–67. doi: 10.1007/s00018-012-1216-x (PMC3607724; doi:10.1007/s00018-012-1216-x)

A

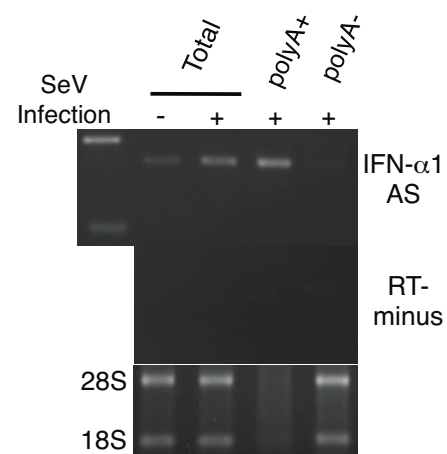

B

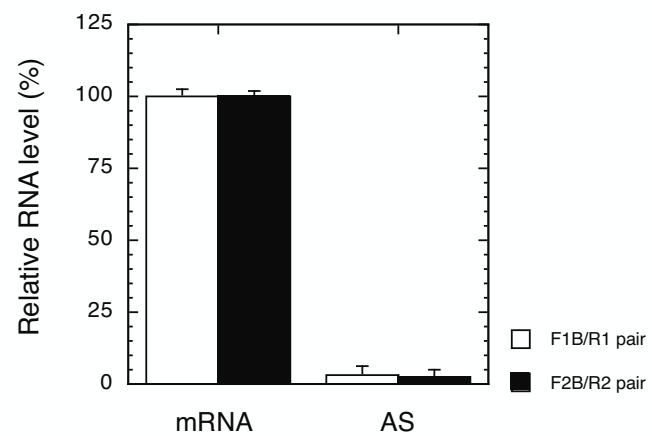

Supplementary Fig.1

Supplement: Supplementary file 2 — Supplementary Fig. 1 Characterisation and quantification of IFN-α1 AS RNA. a To further characterise the AS RNA, total cellular, poly(A)+ and poly(A)- RNAs were extracted and prepared from SeV-infected Namalwa cells 24 h after infection and were analysed by strand-specific RT-PCR using the F1 primer and the F1B/R1 primer pair as described in Materials and methods. 28S and 18S show positions of rRNA bands. RT-minus indicates a negative PCR control without RT. b Quantification of IFN-α1 AS RNA by strand-specific RT-qPCR. Total cellular RNA was prepared from the cells described above. Strand-specific RT was performed for IFN-α1 mRNA (mRNA) and its AS RNA (AS), followed by real-time PCR with the respective primer pairs (□ F1B/R1 or ■ F2B/R2). Values from a representative experiment of three independent infection experiments are shown as the “relative RNA level” ± s.e.m. of triplicate samples, where the level of IFN-α1 mRNA is denoted as 100%. (PDF 73 kb) [file 18_2012_1216_MOESM2_ESM.pdf]

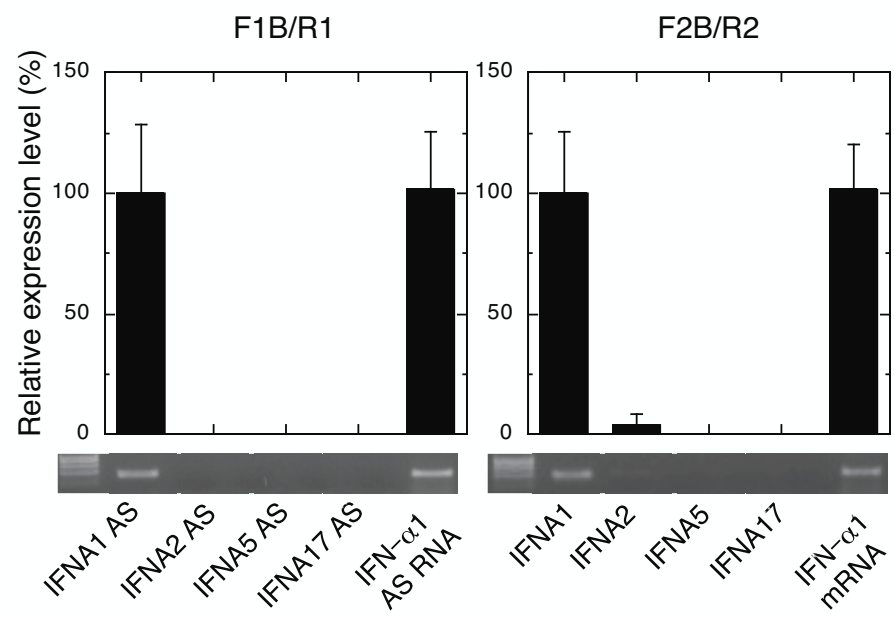

Supplementary Fig.2

Supplement: Supplementary file 3 — Supplementary Fig. 2 Evaluation of the specificity of the real-time PCR assay for the quantification of IFN-α1 AS RNA/mRNA signals. To evaluate the presence of contaminating IFN-α cDNA signals, the F1B/R1 and F2B/R2 primer pairs were tested for amplification of IFNA1, 2, 5 and 17 or their revertants. The amount of IFNA1 plasmid was titrated to produce an antisense cDNA signal very close to that obtained from IFN-α1 AS RNA. This amount was then applied to amplifications using IFNA2, A5 and A17 plasmids. Values from a representative experiment of three independent experiments are shown as the “relative expression level” ± s.e.m. of triplicate samples, where the levels of IFN-α1 AS RNA and IFN-α1 mRNA are denoted as 100%. (PDF 71 kb) [file 18_2012_1216_MOESM3_ESM.pdf]
